# GNL3 Orchestrates AR Transcriptional Programs to Drive Castration‐Resistant Prostate Cancer and Immune Evasion

**DOI:** 10.1002/advs.202516411

**Published:** 2026-03-02

**Authors:** Cuiting Zhang, Tin Long Cheong, Nitin Narwade, Dandan Dong, Mokan Deng, Zhengqiang Miao, Zhaoqiang Ding, Wenchao Li, Kate Man Kei Lei, Gong‐Hong Wei, Terence Chuen Wai Poon, Chu‐Xia Deng, Edwin Cheung

**Affiliations:** ^1^ Cancer Centre University of Macau Taipa Macau SAR China; ^2^ Centre for Precision Medicine Research and Training University of Macau Taipa Macau SAR China; ^3^ MoE Frontiers Science Center for Precision Oncology University of Macau Taipa Macau SAR China; ^4^ Department of Biomedical Sciences Faculty of Health Sciences University of Macau Taipa Macau SAR China; ^5^ Fudan University Shanghai Cancer Center & MOE Key Laboratory of Metabolism and Molecular Medicine Department of Biochemistry and Molecular Biology of School of Basic Medical Sciences Shanghai Medical College of Fudan University Shanghai China; ^6^ Genomics, Bioinformatics & Single Cell Core Faculty of Health Sciences University of Macau Taipa Macau SAR China; ^7^ Department of Urology Zhongda Hospital Southeast University Nanjing Jiangsu China; ^8^ Pilot Laboratory University of Macau Taipa Macau SAR China; ^9^ Institute of Translational Medicine University of Macau Taipa Macau SAR China; ^10^ State Key Laboratory of Common Mechanism Research for Major Diseases Suzhou Institute of Systems Medicine Chinese Academy of Medical Sciences & Peking Union Medical College Suzhou Jiangsu China; ^11^ Zhuhai UM Science & Technology Research Institute (ZUMRI), Hengqin Zhuhai China; ^12^ Present address: Instituto de Neurociencias (CSIC‐UMH) Zhuhai Spain

**Keywords:** AR coregulator, castration‐resistant prostate cancer, GNL3, immunosuppression

## Abstract

Androgen receptor (AR) signaling is a primary oncogenic driver of castration‐resistant prostate cancer (CRPC), yet the mechanism remains incompletely understood. Through proteomic profiling of CRPC and primary PCa cells, we identify G Protein Nucleolar 3 (GNL3) as a novel AR coregulator. GNL3 physically interacts with AR, enhances its chromatin occupancy, and directly coactivates transcriptional programs that promote cell proliferation, including NEK2 and CDC20. Concurrently, GNL3 functions as a corepressor of immune‐responsive genes such as CXCL10 and TAP1 via class I histone deacetylases (HDACs), thereby facilitating CD8+ T cell elimination and establishing an immunosuppressive tumor microenvironment. GNL3 expression and AR‐GNL3 complex formation progressively increase from normal prostate to CRPC and correlate with poor clinical outcomes. Functionally, GNL3 knockdown sensitizes CRPC cells to AR antagonists and impairs tumor growth and metastasis. Furthermore, we demonstrate that combinatorial inhibition of NEK2, class I HDACs, and AR signaling can be a potential therapeutic strategy for CRPC. Overall, these findings establish GNL3 as a dual‐function AR coregulator and therapeutic target, providing mechanistic insights into transcriptional regulation and immune evasion in advanced PCa.

## Graphical abstract

GNL3 is a novel AR coregulator with dual coactivator and corepressor functions in prostate cancer (PCa). Our study uncovers a previously unrecognized mechanism by which the AR transcriptional complex integrates oncogenic signaling and immune suppression. Targeting GNL3, either directly or through rational combination therapies, represents a promising strategy to overcome therapeutic resistance and improve treatment outcomes in advanced PCa.

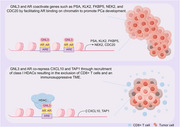

## 1 Introduction

Prostate cancer (PCa) is the second most commonly occurring cancer in men globally. Androgen receptor (AR) signaling plays a pivotal role in the initiation and progression of PCa [1]. Although androgen deprivation therapy (ADT), through surgical or chemical castration, remains the frontline standard of care for primary PCa and significantly enhances patient survival [2], most patients eventually relapse, acquiring resistance and developing castration‐resistant prostate cancer (CRPC), a highly aggressive and fatal stage of PCa. Despite androgen ablation, AR signaling is frequently reactivated in CRPC, as evidenced by elevated serum prostate‐specific antigen (PSA), a well‐established AR target and clinical diagnostic marker [3].

Multiple mechanisms contribute to AR reactivation in CRPC, including gene amplification, gain‐of‐function mutations, aberrant post‐translational modifications, and the expression of AR variants, which results in constitutive activation of AR [4, 5]. Intracrine androgen synthesis in tumors also contributes to sustained AR signaling in CRPC [6]. Beyond these well‐characterized mechanisms, dysregulation of the AR co‐regulatory network has emerged as a key driver of castration resistance [7, 8, 9, 10, 11, 12]. For instance, SRC‐1, an AR coactivator, has been shown to promote CRPC cell growth and alter AR target gene expression [8]. However, many AR coregulators remain unidentified in CRPC, and their roles in CRPC are poorly understood.

AR‐mediated transcription requires a coordinated assembly of cofactors at AR binding sites (ARBSs) [10, 11, 12]. Cofactors are broadly categorized into three groups. First, pioneer factors such as FOXA1 remodel chromatin to facilitate AR DNA binding to specific genomic loci [13]. Second, coregulators include coactivators like CBP/p300 and corepressors such as NCoR, SMRT, HDAC, EZH2, and LSD1, which are non‐DNA‐binding proteins and fine‐tune AR transcriptional activity [14, 15, 16, 17]. For instance, CBP/p300 enhances AR‐dependent transcription by bridging enhancers to RNA polymerase II and acetylating histones [14, 15], while corepressors attenuate AR transcriptional activity by chromatin compaction and transcriptional repression [16, 17]. Third, collaborative factors such as ERG facilitate the recruitment of AR transcriptional complexes [11]. Dissecting the composition and function of these cofactors is essential to understanding AR‐driven oncogenic programs and therapeutic resistance.

Emerging evidence indicates that an immunologically “cold” tumor microenvironment (TME) contributes to CRPC progression [18, 19, 20]. PCa generally displays low T cell infiltration and responds poorly to immune checkpoint blockade therapies [21]. More aggressive subtypes, such as CRPC and NEPC, exhibit even lower CD8+ T cell presence compared to benign prostatic hyperplasia (BPH) and primary PCa [18, 19, 20]. Recent mechanistic studies have implicated AR signaling in shaping this immunosuppressive landscape. Specifically, AR suppressed interferon signaling in T cells and MHC Class I expression in tumor cells, leading to immune evasion and resistance to immunotherapy in PCa [22, 23]. Additionally, AR activation in macrophages induced IL‐1β secretion, driving the accumulation of myeloid‐derived suppressor cells (MDSCs) and suppressing CD8+ T cell activity [24]. While targeting oncogenic pathways like PI3K signaling or chromatin regulators such as Pygo2 have shown promise in reprogramming the TME and enhancing anti‐immunity [25, 26], the role of AR coregulators in modulating immune responses remains largely unexplored.

In this study, we identify G Protein Nucleolar 3 (GNL3) as a novel AR coregulator through proteomics‐based rapid immunoprecipitation of endogenous proteins (RIME) [27] analysis in CRPC cells. We show that GNL3 is clinically upregulated in PCa, with peak expression in CRPC, and its levels correlate with poor patient prognosis. Integration of molecular, genomic, and single‐cell analysis, including imaging mass cytometry (IMC), reveals that GNL3 and AR cooperatively regulate transcriptional programs that simultaneously promote cell proliferation and repress immune‐responsive genes to promote an immunosuppressive PCa TME. Finally, we demonstrate that combined inhibitions of NEK2, class I HDACs, and AR significantly enhance anti‐tumor efficacy in vivo, highlighting promising therapeutic strategies for CRPC.

## 2 Results

### 2.1 High GNL3 Expression is Associated with CRPC Progression and Poor Prognosis

To identify AR‐interacting proteins linked to CRPC progression, we performed RIME analysis targeting AR in the CRPC cell line C4‐2B. This analysis captured 455 AR‐associated proteins (Figure S1A), including well‐known AR cofactors such as FOXA1, NKX3‐1, p300, NCoR1, NCoR2, HDAC1, and HDAC2, consistent with our previous findings in LNCaP and VCaP cells [28]. A comparison across the three cell lines revealed 86 shared AR interactors (Figure S1A and Table S1).

**FIGURE 1:**
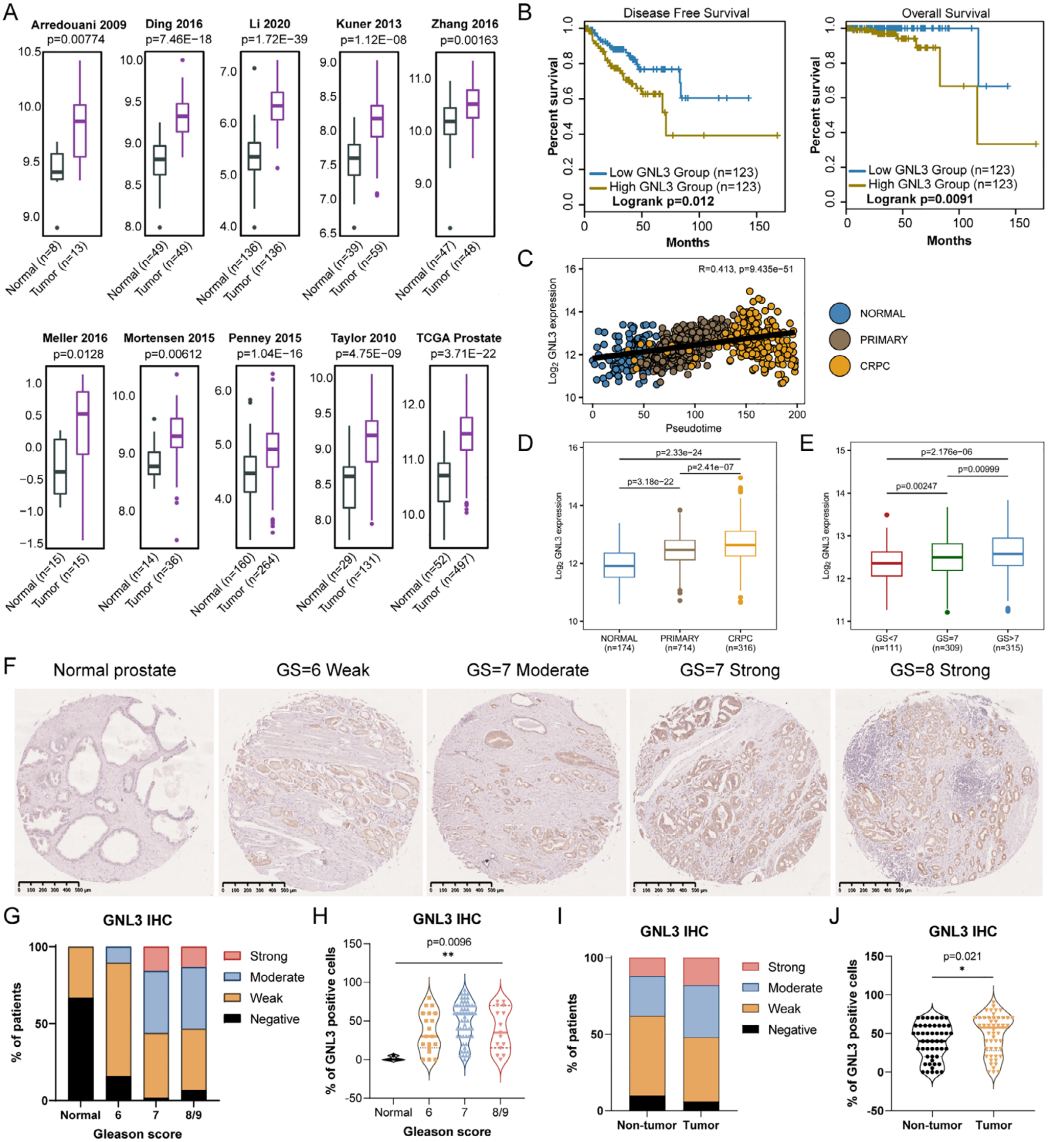
GNL3 is highly expressed in PCa and associated with unfavorable clinical outcomes (A) Boxplots showing GNL3 expression levels in normal vs. tumor tissues across multiple PCa patient cohorts. *p*‐value: Mann‐Whitney U test. (B) Kaplan‐Meier analysis depicting disease‐free and overall survival in TCGA‐PRAD cohorts stratified by top 25% (high) and bottom 25% (low) GNL3 expression groups. *p*‐value: log‐rank test. (C) Scatterplot showing the correlation between GNL3 expression and PCa progression in the PCaProfiler dataset (*n* = 1,204). Variance stabilizing transformation (VST)‐normalized GNL3 expression was correlated with pseudotime trajectory scores. Pearson correlation and linear regression are shown. (D) Boxplot comparing GNL3 expression in normal prostate, primary PCa, and CRPC samples from C. *p*‐value: Mann‐Whitney U test. (E) GNL3 expression across Gleason score (GS) groups. *p*‐value: Mann‐Whitney U test. (F) Representative IHC images of GNL3 staining in a human PCa tissue microarray. Scale bar: 500 µm. (G), (H) Quantification of GNL3 staining intensity and percentage of positive cells in different GS groups. *p*‐value: Kruskal‐Wallis test. (I), (J) Comparison of GNL3 intensity and positive cells percentage between tumor and non‐tumor tissues. *p*‐value: Mann‐Whitney U test.

To prioritize candidates for further study, we conducted a literature review and identified 31 proteins with limited prior characterization in PCa. We then analyzed their expression and prognostic relevance using publicly available clinical datasets. Among these, GNL3 emerged as one of the most promising candidates, exhibiting consistently higher expression across multiple datasets and a strong correlation with poor patient survival. Analysis of multiple PCa datasets [29, 30, 31, 32, 33, 34, 35, 36, 37] revealed that GNL3 expression is significantly higher in tumors compared to normal tissues (Figure 1A). Moreover, patients with high GNL3 levels exhibited poorer disease‐free and overall survival (Figure 1B). To further investigate the relationship between GNL3 expression and disease progression, we performed pseudotime trajectory analysis on 1,204 clinical PCa specimens from the integrated PCaProfiler dataset [38]. GNL3 expression increased progressively from normal prostate to primary PCa, peaking in CRPC (Figure 1C). Stratified analysis confirmed significantly higher GNL3 expression in primary tumors relative to normal tissue, with further elevation in CRPC (Figure 1D). Additionally, GNL3 levels positively correlated with Gleason score, increasing from low‐ to high‐grade tumors (Figure 1E).

To examine GNL3 protein expression in PCa patient samples, we performed immunohistochemistry (IHC) on a tissue microarray (TMA) comprising tumor and non‐tumor prostate tissues. GNL3 staining intensity was markedly higher in tumor tissues and increased progressively with Gleason grade (Figure 1F,G), mirroring transcriptomic patterns observed in PCaProfiler (Figure 1E). In addition, the proportion of GNL3‐positive cells exhibited a marked increase in tumor tissues relative to normal prostate (Figure 1H). Comparison of tumor and non‐tumor tissues further confirmed elevated GNL3 staining and increased GNL3‐positive cell counts in tumors (Figure 1I,J; Figure S1B). Together, these data establish GNL3 as a clinically relevant AR interactor that is upregulated during PCa progression, particularly CRPC, and strongly associated with unfavorable clinical outcomes. Furthermore, these results suggest a pro‐tumorigenic function for GNL3 and underscore its potential utility as a biomarker and therapeutic target in advanced PCa.

### 2.2 GNL3 is Essential for Tumor Growth and Metastasis in CRPC

**FIGURE 2:**
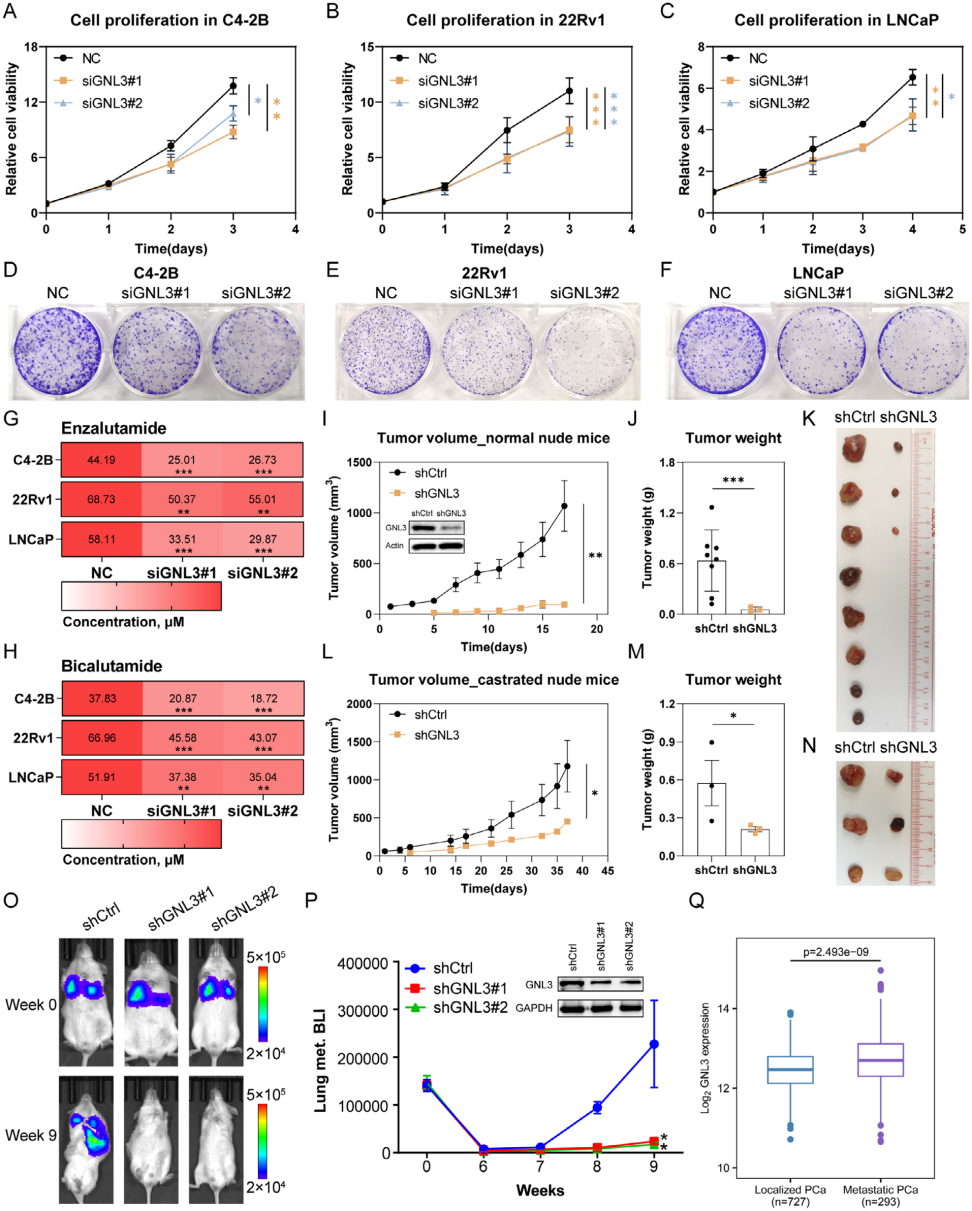
GNL3 is essential for tumor growth and metastasis in CRPC (A–C) Cell proliferation in C4‐2B (A), 22Rv1 (B), and LNCaP (C) cells following GNL3 knockdown. Error bars present ± SD (*n* = 3). *p*‐value: two‐way ANOVA. (D–F) Colony formation assays in C4‐2B (D), 22Rv1 (E), and LNCaP (F) upon GNL3 knockdown. (G), (H) IC_50_ values of enzalutamide (G) and bicalutamide (H) in C4‐2B, 22Rv1, and LNCaP cells with or without GNL3 knockdown. *p*‐value: two‐way ANOVA. (I–N) Tumor growth of C4‐2B xenografts in intact (I–K) and castrated nude mice (L–N). GNL3 knockdown (shGNL3) and control (shCtrl) cells were inoculated into the flanks of intact or castrated male nude mice. Tumor growth and tumor weight are shown. Tumors were excised at the endpoint (K, *n* = 8 and N, *n* = 3). Error bars present ± SEM. *p*‐value: two‐way ANOVA. (O), (P) Bioluminescence imaging (BLI) of lung metastases in NOD‐SCID mice intravenously injected with luciferase‐labeled 22Rv1 cells stably expressing shCtrl or shGNL3. Quantification over 9 weeks shows a marked and statistically significant reduction in metastatic tumor burden in the GNL3 knockdown group. Error bars present ± SD (*n* = 3). *p*‐value: two‐sided Student's *t*‐test. (Q) Boxplot showing GNL3 expression level in localized PCa and metastatic PCa in the PCaProfiler dataset. *p*‐value: Mann‐Whitney U test. (^***^ *p* < 0.001; ^**^ *p* < 0.01; ^*^ *p* < 0.05; ns, not significant at the 0.05 level).

To elucidate the functional significance of GNL3 in PCa, we performed targeted knockdown of GNL3 in hormone‐sensitive (LNCaP) and castrate‐resistant (C4‐2B and 22Rv1) PCa cell lines. Silencing GNL3 significantly impaired cell proliferation across all three cell line models (Figure 2A–C; Figure S2A–C). Consistently, colony formation assays revealed a marked reduction in clonogenic potential following GNL3 depletion (Figure 2D–F). Given the widespread clinical use of AR antagonists and the frequent emergence of therapeutic resistance, we next examined whether GNL3 knockdown could sensitize PCa cells to AR inhibition. Co‐treatment with GNL3 siRNA and FDA‐approved AR antagonists, enzalutamide or bicalutamide, resulted in marked inhibition of cellular proliferation and reduced the half‐maximal inhibitory concentration (IC_50_) for both drugs (Figure 2G,H; Figure S2D), suggesting that GNL3 depletion enhances therapeutic response.

To determine the role of GNL3 in prostate tumor growth in vivo, we established C4‐2B cell lines stably expressing GNL3‐targeting shRNA or a non‐targeting control (shCtrl) and transplanted them subcutaneously into both intact (Figure 2I–K) and surgically castrated male nude mice (Figure 2L–N; Figure S2E). In both models, GNL3 knockdown led to a marked reduction in tumor volume and weight compared to controls, indicating a crucial role for GNL3 in supporting tumor growth irrespective of androgen status.

We next examined whether GNL3 contributes to PCa metastasis. In vitro migration and invasion assays demonstrated that GNL3 depletion markedly impaired the migratory and invasive capacity of PCa cells, supporting a role for GNL3 in promoting PCa metastasis (Figure S2F, G). To assess its contribution in vivo, we injected 22Rv1 cell lines stably expressing firefly luciferase into the tail veins of NOD‐SCID mice and monitored tumor burden in the lungs via bioluminescent imaging (BLI). Mice injected with GNL3‐depleted cells exhibited a dramatic reduction in lung colonization compared to controls, as evidenced by significantly lower BLI signals (Figure 2O,P). In support of this observation, analysis of patient samples from PCaProfiler showed GNL3 expression is elevated in metastatic PCa compared to localized PCa (Figure 2Q; Figure S2H). Collectively, these results identify GNL3 as a critical driver of tumor growth and metastasis in hormone‐sensitive PCa and CRPC, highlighting its potential as a therapeutic target across various stages of disease progression.

### 2.3 GNL3 Interacts with AR in PCa Cells

**FIGURE 3:**
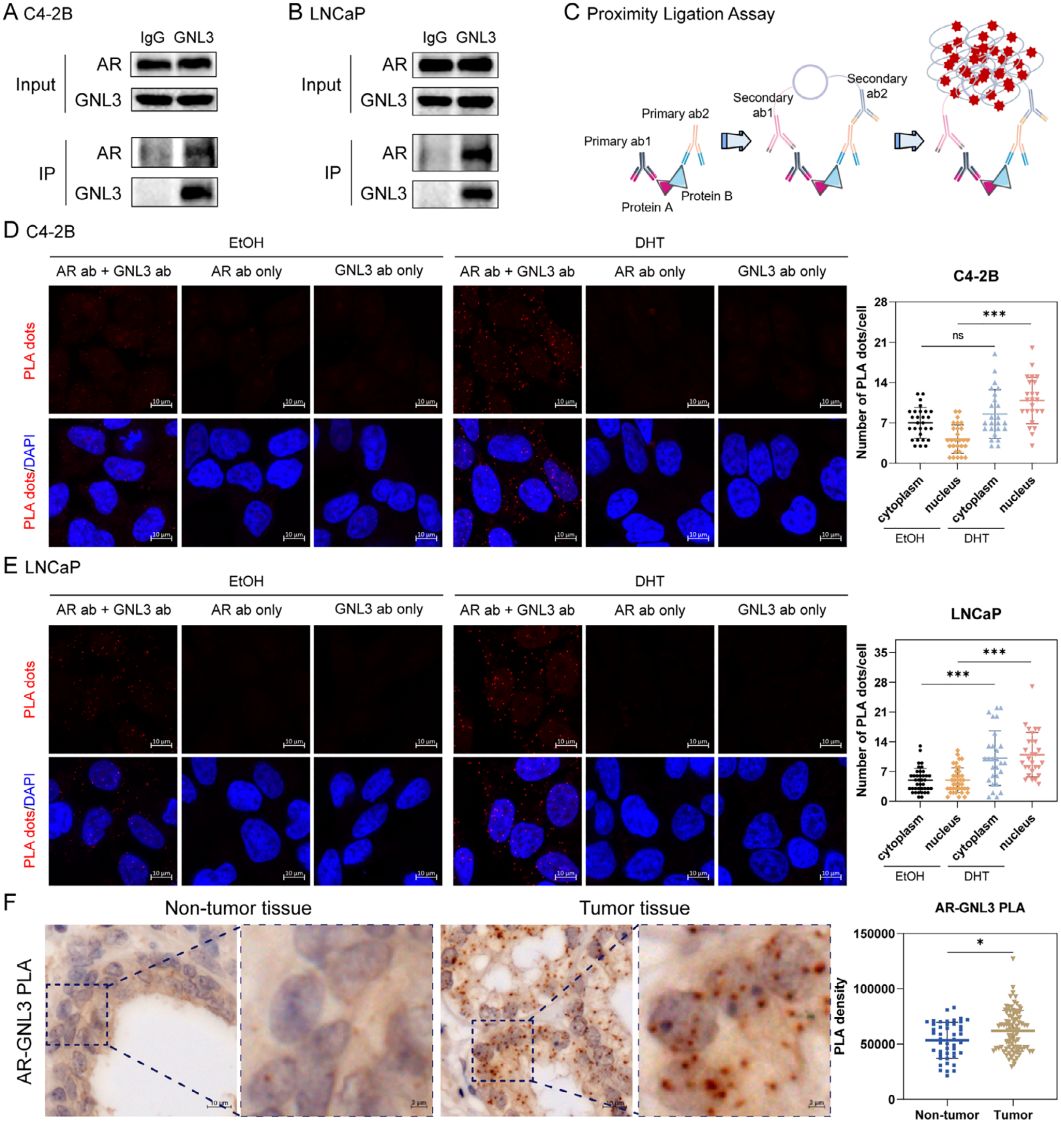
GNL3 is a novel AR co‐regulator (A), (B) Co‐immunoprecipitation of GNL3 and AR in C4‐2B (A) and LNCaP (B) cells. IgG was used for the negative control. (C) A schematic diagram of the proximity ligation assay (PLA). Antibody, ab. (D), (E) Representative PLA images showing AR‐GNL3 interaction in C4‐2B (D) and LNCaP (E) cells under 2 h EtOH or DHT treatment. Red fluorescence dots indicate protein‐protein interaction. *p*‐value: Mann‐Whitney U test. (F) Quantification of AR‐GNL3 PLA signals in tumor vs. non‐tumor tissues from the human PCa TMA. Quantifications of PLA dots were performed using the ZEN microscopy software. *p*‐value: Mann‐Whitney U test. (^***^ *p* < 0.001; ^**^ *p* < 0.01; ^*^ *p* < 0.05; ns, not significant at the 0.05 level).

To delineate the mechanistic contribution of GNL3 in PCa, we first assessed its interaction with AR. Co‐immunoprecipitation assays in C4‐2B and LNCaP cells confirmed the physical interaction between GNL3 and AR, validating our RIME findings (Figure 3A,B). To further substantiate this interaction, we conducted in situ proximity ligation assays (PLA; Figure 3C) in PCa cell lines and patient samples on the TMA. PLA signals, indicative of protein‐protein interaction, were observed under both EtOH and DHT conditions when AR and GNL3 primary antibodies were used (Figure 3D,E). These signals were absent in single antibody controls, confirming the specificity of the assay. Both nuclear and cytoplasmic PLA signals were detected, consistent with the subcellular localization of GNL3 and AR (data not shown). Notably, DHT significantly enhanced nuclear PLA signals, suggesting androgen signaling promotes AR‐GNL3 complex formation. In patient samples, PLA signals were significantly enriched in tumor compared to non‐tumor tissues (Figure 3F), indicating that AR‐GNL3 interactions are more frequent in the malignant state.

### 2.4 GNL3 Colocalizes with AR on Chromatin and Facilitates AR‐Driven Transcription

**FIGURE 4:**
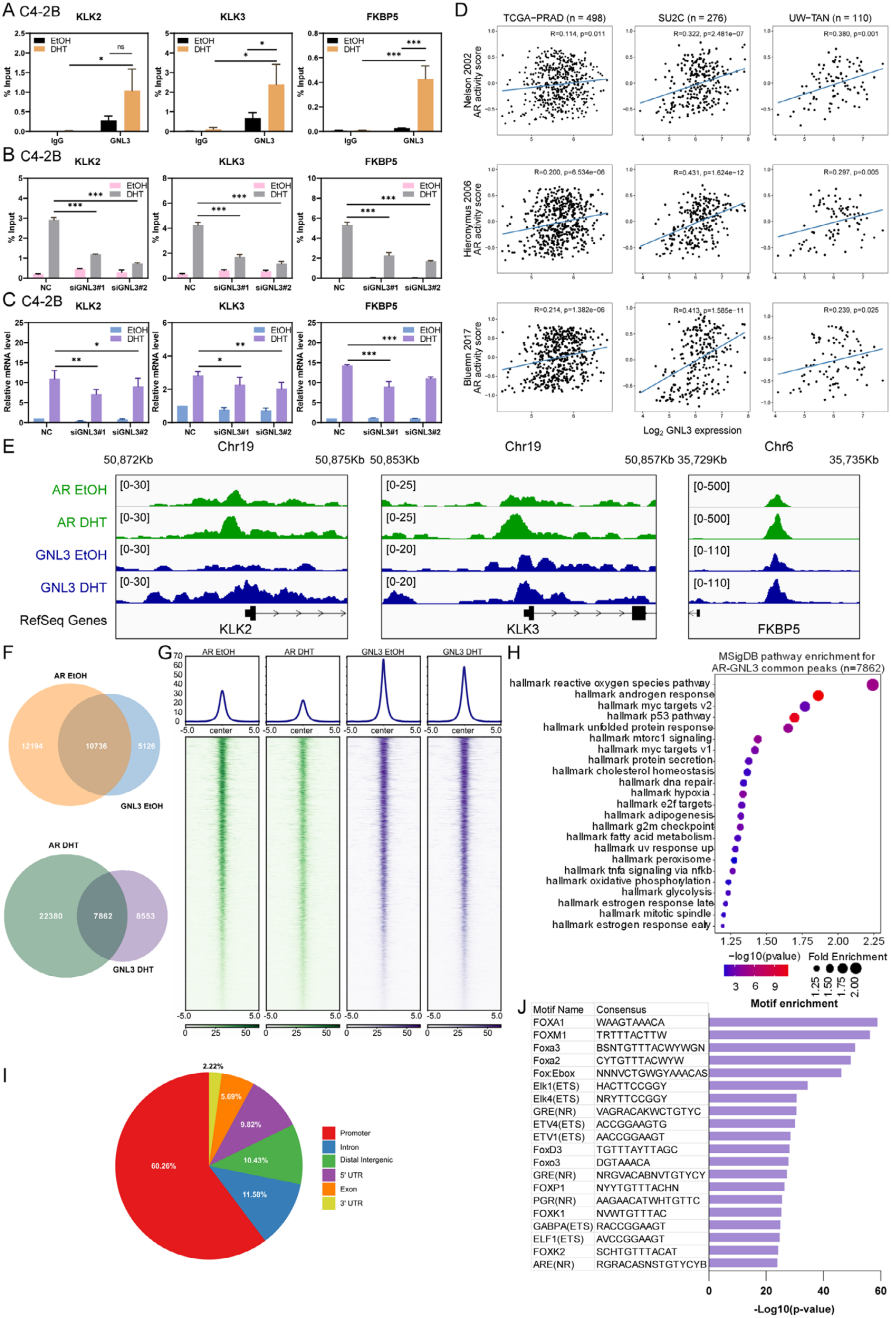
GNL3 colocalizes with AR on chromatin and is required for AR‐activated transcription (A) GNL3 ChIP‐qPCR at AR target genes (KLK2, KLK3, and FKBP5) in C4‐2B cells with or without DHT treatment (2 h). Data are shown as a percentage of input. IgG was used for the negative control. Error bars present ± SEM (*n* = 3). *p*‐value: two‐way ANOVA. (B) AR ChIP‐qPCR after GNL3 knockdown at KLK2, KLK3, and FKBP5 genes in C4‐2B under 2 h EtOH or DHT treatment. Data are shown as a percentage of input. Error bars present ± SEM (*n* = 3). *p*‐value: two‐way ANOVA. (C) RT‐qPCR analysis of KLK2, KLK3, and FKBP5 expression in C4‐2B cells following GNL3 knockdown. Error bars present ± SD (*n* = 3). *p*‐value: two‐way ANOVA. (D) Correlation between GNL3 expression and AR‐activated gene signatures in human PCa datasets. Pearson correlation coefficient was calculated, and the associated p‐value was used. (E) Genomic tracks of AR and GNL3 binding peaks through ChIP‐seq analysis around AR targets (KLK2, KLK3, and FKBP5) in C4‐2B cells under 2 h EtOH or DHT treatment. (F) Venn diagram illustrating the intersection of AR and GNL3 binding peaks through ChIP‐seq analysis in C4‐2B cells. (G) Average tag densities (upper panel) of AR and GNL3 ChIP‐seq and heatmaps (lower panel) of shared AR and GNL3 ChIP‐seq peaks. Data are shown as normalized binding intensity. (H) MSigDB pathway enrichment analysis of genes associated with AR and GNL3 co‐occupied peaks. (I) Pie chart showing genomic distribution of AR‐GNL3 co‐occupied peaks. (J) Bar plot of the top 20 enriched motifs in AR‐GNL3 shared peaks. (^***^ *p* < 0.001; ^**^ *p* < 0.01; ^*^ *p* < 0.05; ns, not significant at the 0.05 level).

We next investigated whether GNL3 is recruited to AR target genes and modulates AR‐dependent transcription. ChIP assays demonstrated that GNL3 binds to AR binding sites (ARBSs) of canonical AR‐activated genes, including KLK2, KLK3, and FKBP5, in both C4‐2B and LNCaP cells (Figure 4A; Figure S3A). DHT treatment significantly enhanced GNL3 occupancy at these sites, suggesting that its recruitment is androgen dependent. To determine whether GNL3 binding requires AR, we performed GNL3 ChIP following AR knockdown. Loss of AR substantially reduced GNL3 occupancy on these ARBSs (Figure S3B), indicating that AR is necessary for GNL3 binding. Conversely, AR ChIP performed after GNL3 knockdown showed AR binding at these target genes was significantly decreased in GNL3‐depleted cells (Figure 4B; Figure S3C), suggesting that GNL3 is required for efficient AR chromatin engagement.

To determine whether GNL3 directly regulates AR‐dependent transcription, we performed RT‐qPCR on AR‐activated genes upon GNL3 knockdown. GNL3 depletion led to reduced expression levels of KLK2, KLK3, and FKBP5 (Figure 4C; Figure S3D, E), indicating that GNL3 is required for AR‐activated transcription. Supporting these findings, analysis of publicly available datasets showed a positive association between GNL3 levels and AR‐activated gene signatures [39, 40, 41] or AR expression level in both primary PCa and CRPC patient cohorts (Figure 4D; Figure S3F). Together, our results reveal that GNL3 coregulates AR‐activated transcription by facilitating AR binding on chromatin in hormone‐sensitive and castration‐resistant PCa.

To define the global chromatin landscape of GNL3 and its relationship with AR, we performed ChIP‐seq analysis for GNL3 and AR in C4‐2B cells. Consistent with our ChIP‐qPCR results, GNL3 colocalized with AR at regulatory regions of KLK2, KLK3, and FKBP5 (Figure 4E). In total, we identified 22,930 ARBSs and 15,862 GNL3 binding sites (GNL3BSs) under EtOH treatment and 30,242 ARBSs and 16,415 GNL3BSs under DHT stimulation (Figure 4F). Notably, 10,736 sites overlapped under EtOH conditions and 7,862 under DHT, indicating substantial co‐occupancy of GNL3 and AR across the genome (Figure 4F,G; Table S2).

Functional annotation of AR‐GNL3 co‐binding sites revealed enrichment for genes involved in key oncogenic pathways, including androgen response, MYC targets, p53 pathway, DNA repair, mTORC1 signaling, and cell cycle regulation (Figure 4H). Interestingly, genomic distribution analysis showed that AR‐GNL3 co‐occupancy was predominantly localized to promoter regions (60.26%) (Figure 4I), which is distinct from enhancer‐centric AR coregulators such as CBP/p300 [42]. Moreover, motif analysis identified significant AR response element (ARE) enrichment among shared binding sites (Figure 4J), further supporting direct recruitment of GNL3 to AR‐bound chromatin. Together, these findings establish GNL3 as a chromatin‐associated AR coregulator that facilitates AR binding and transcriptional activation, primarily at promoter‐proximal regions in PCa cells.

### 2.5 NEK2 and CDC20 Mediate GNL3‐Driven Transcriptional Programs and Synergize with AR Inhibition in PCa

**FIGURE 5:**
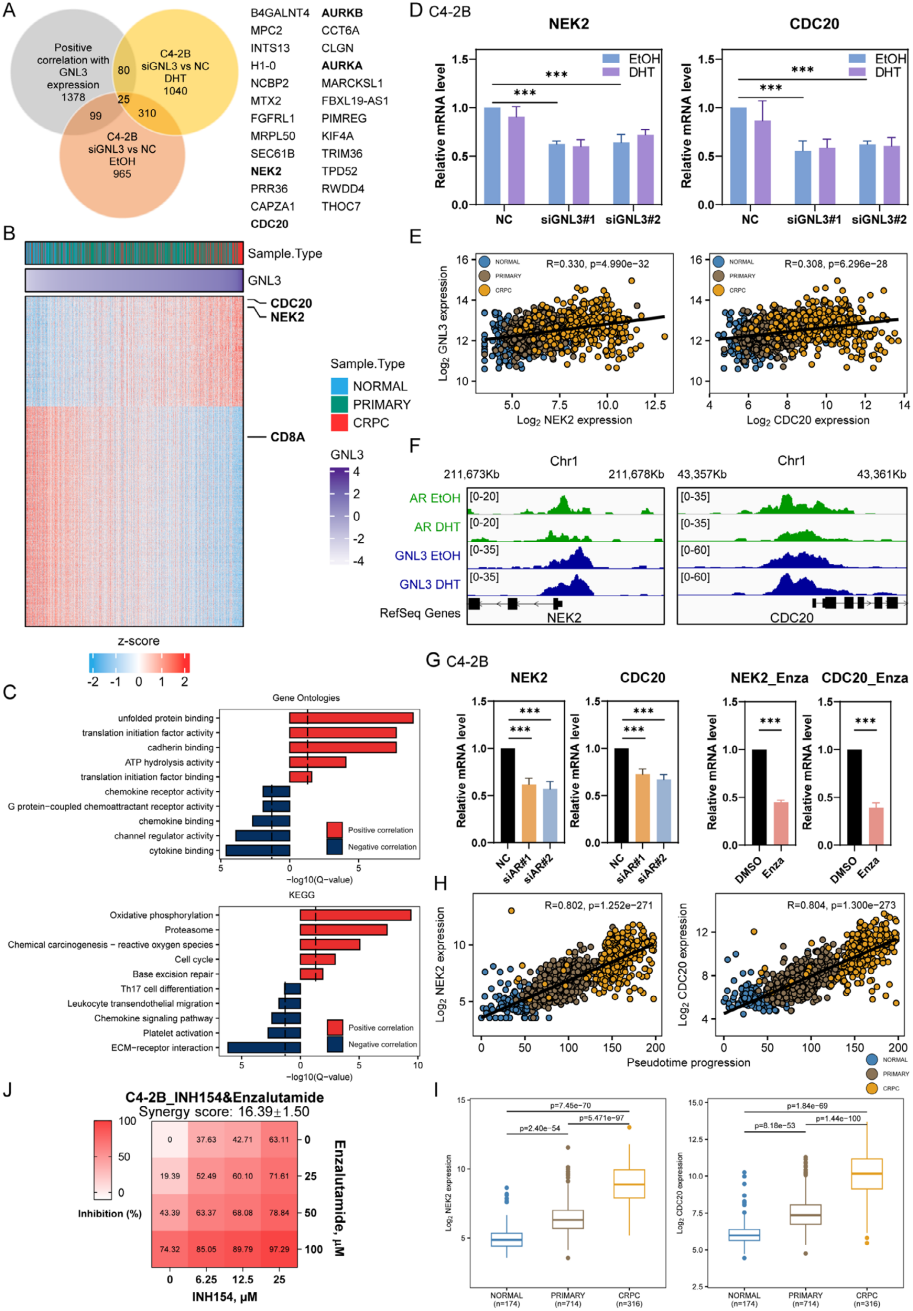
NEK2 and CDC20 mediate GNL3‐driven transcriptional programs and synergize with AR inhibition in PCa (A) Venn diagram illustrating the intersection of genes positively correlated with GNL3 in human PCa (from panel B) and GNL3 upregulated genes from RNA‐seq analysis following GNL3 knockdown in C4‐2B cells. (B) Heatmap representing the relative expression of genes significantly correlated with GNL3 (Pearson ≥ 0.3, Pearson ≤ ‐0.3) in the PCaProfiler dataset. Z‐scores were calculated from batch‐corrected VST‐normalized expression. Subtype classification and GNL3 expression levels are shown above the heatmap. (C) Bar plots showing enrichment of biological processes by Gene Ontologies (upper panel) and KEGG pathways (lower panel) of positively and negatively correlated genes with GNL3 (from panel B). (D) RT‐qPCR analysis of NEK2 and CDC20 mRNA expression in C4‐2B cells following GNL3 knockdown. Error bars present ± SD (*n* = 3). *p*‐value: two‐way ANOVA. (E) Scatterplot illustrating the correlation between GNL3 and NEK2/CDC20 expression in the PCaProfiler dataset. Pearson correlation coefficient was calculated, and the associated p‐value was used. (F) Genomic tracks of AR and GNL3 ChIP‐seq peaks around NEK2 and CDC20 in C4‐2B cells. (G) RT‐qPCR analysis of NEK2 and CDC20 expressions in C4‐2B cells following AR knockdown or enzalutamide treatment. Error bars present ± SD (*n* = 3). *p*‐value: two‐way ANOVA. (H) Scatterplot showing correlation between pseudotime trajectory and NEK2/CDC20 expression in the PCaProfiler dataset. Pearson correlation coefficient was calculated, and the associated p‐value was used. (I) Boxplot showing GNL3 expression across PCa subtypes in the PCaProfiler dataset. *p*‐value: Mann‐Whitney U test. (J) Heatmap showing percentage of inhibition on cell proliferation in C4‐2B cells treated with NEK2 inhibitor INH154 and enzalutamide. Synergy scores were calculated using the HSA method in SynergyFinder. (^***^ *p* < 0.001; ^**^ *p* < 0.01; ^*^ *p* < 0.05; ns, not significant at the 0.05 level).

To define the transcriptional programs orchestrated by GNL3 and identify potential therapeutic targets in PCa, we first performed a correlation analysis between GNL3 expression and transcriptomic profiles from the PCaProfiler clinical dataset [38]. This analysis revealed 1,378 genes positively correlated and 2,751 genes negatively correlated with GNL3 expression (Figure 5A,B). Functional enrichment analysis of positively correlated genes highlighted pathways central to PCa progression [43, 44, 45], including cell cycle regulation, translation, and MYC targets. In contrast, negatively correlated genes were highly enriched in chemokine binding, chemokine signaling, and p53 pathways (Figure 5C; Figure S4A).

To complement these clinical findings, we conducted RNA‐seq analysis in C4‐2B cells following GNL3 knockdown. We identified 965 and 1,040 GNL3‐activated genes under EtOH and DHT conditions, respectively (Figure 5A; Table S3). Intersecting GNL3‐upregulated genes with those positively correlated in clinical samples yielded 25 candidate effectors, including NEK2, CDC20, AURKA, and AURKB (Figure 5A). Since previous studies have already demonstrated the efficacy of combining AURKA and AURKB inhibitors with AR blockade in PCa [46, 47, 48, 49], we focused our efforts on NEK2 and CDC20 as novel, GNL3‐regulated therapeutic targets.

We first examined the transcriptional regulation of NEK2 and CDC20 by GNL3. GNL3 knockdown significantly downregulated NEK2 and CDC20 expression in both C4‐2B and LNCaP cells (Figure 5D; Figure S4B), and their expression levels positively correlated with GNL3 in both primary and CRPC samples (Figure 5E). ChIP assays confirmed co‐occupancy of GNL3 and AR at the promoter regions of NEK2 and CDC20, indicating direct transcriptional co‐regulation of these genes by GNL3 and AR (Figure 5F; Figure S4C, D). Furthermore, we assessed their mRNA levels in C4‐2B and LNCaP cells following AR inhibition via siRNA or treatment with AR inhibitor enzalutamide. Both approaches significantly reduced NEK2 and CDC20 expressions (Figure 5G; Figure S4E, F), further supporting their co‐regulation by GNL3 and AR. Importantly, NEK2 and CDC20 expression levels increased progressively from normal prostate tissue to primary PCa, peaking in CRPC (Figure 5H,I), suggesting a critical role for these factors in disease progression.

To assess the therapeutic potential of targeting NEK2 and CDC20, we evaluated the effects of their pharmacological inhibition with AR inhibitors on PCa cell proliferation. C4‐2B and LNCaP cells were treated with the NEK2 inhibitor INH154 or the CDC20 inhibitor Apcin, either alone or in combination with AR inhibitors enzalutamide or bicalutamide. Drug synergy was quantified using the HSA synergy score in SynergyFinder [50], where a score above 10 indicates synergy and a score between −10 and 10 indicates additivity (Figure 5J; Figure S4G,H). Notably, INH154 exhibited greater potency and a higher synergy score than Apcin in both cell lines. Together, these findings reveal that the GNL3 drives a transcriptional program promoting PCa progression through NEK2 and CDC20, and that dual targeting of these effectors alongside AR inhibition offers a promising therapeutic strategy for advanced PCa.

### 2.6 GNL3 Represses Immune‐Responsive Genes to Eliminate CD8+ T Cells, Driving an Immunosuppressive TME in PCa

**FIGURE 6:**
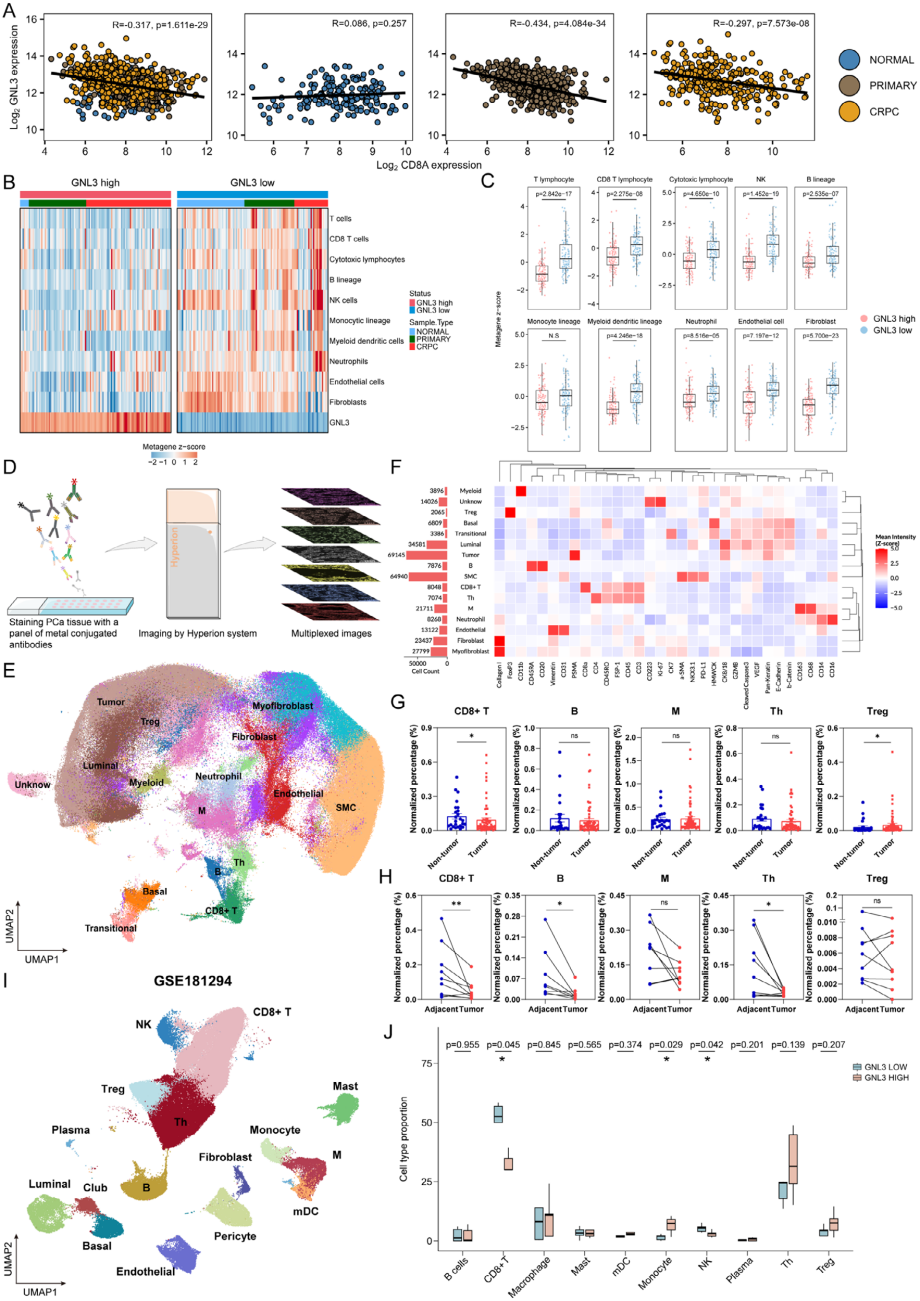
GNL3 promotes CD8+ T cell exclusion and drives an immunosuppressive TME (A) Scatterplot showing correlation between GNL3 and CD8A expression across all samples and stratified by normal, primary PCa only, and CRPC only in the PCaProfiler dataset. Pearson correlation coefficient was calculated, and the associated p‐value was used to determine statistical significance. (B), (C) MCP‐counter immune deconvolution analysis comparing GNL3 high and GNL3 low samples in the PCaProfiler dataset. The MCP‐counter abundance score was transformed through z‐score. Statistical significance was assessed using a two‐sided Student's *t*‐test. (D) A schematic diagram of the IMC analysis. (E) Uniform manifold approximation and projection (UMAP) plot showing IMC analysis from the PCa TMA. Cells are colored based on their cell type annotation according to a panel of 32 antibodies. (F) Heatmap illustrating the expression level of markers in each cluster in D. The left bar plot represents the cell count of each cluster. The expression level of markers is indicated by mean intensity (z‐score). (G) Bar plot showing percentages of immune cell types in tumor vs. non‐tumor regions from IMC analysis. *p*‐value: Mann‐Whitney U test. (H) Paired dot plots from the IMC analysis comparing immune cell percentages in GNL3‐high tumor regions vs. adjacent GNL3‐low non‐tumor regions. *p*‐value: Wilcoxon signed‐rank test. (I) UMAP visualization of single‐cell RNA‐seq data (GSE181294) from 31 PCa samples. Cells are colored based on their cell type annotation. (J) Boxplots showing immune cell lineage proportions in GNL3‐high vs. GNL3‐low PCa patients. *p*‐value: two‐sided Student's *t*‐test. (^***^ *p* < 0.001; ^**^ *p* < 0.01; ^*^ *p* < 0.05; ns, not significant at the 0.05 level).

PCa is widely recognized as an immunologically “cold” tumor, but the mechanisms underlying immune exclusion remain poorly understood. To investigate whether GNL3 contributes to immunomodulation in PCa, we first examined its correlation with immune cell markers. Notably, GNL3 expression was negatively correlated with CD8A (Figure 6A), a key functional marker for CD8+ T cells [51, 52], in both primary PCa and CRPC, but not in normal prostate tissue (Figure 6A). Similar inverse correlations were observed with CD3 subunits (CD3E and CD3G) and CD69, a marker for activated CD8+ T cells (Figure S5A). Consistent with previous reports [18, 19, 20], expression of these markers declined significantly with advancing disease stage (Figure S5B). Together, these data suggest that GNL3 may contribute to immune suppression during PCa progression.

Next, we evaluated the potential influence of GNL3 on the PCa TME by conducting an immune deconvolution analysis using MCP‐counter [53] across normal, primary PCa, and CRPC samples. Stratifying samples by GNL3 expression revealed that GNL3‐high tumors were associated with a markedly immunosuppressive TME (Figure 6B,C). In the normal prostate, GNL3 expression did not significantly affect immune cell populations (Figure S5C, D, I). However, in primary PCa and CRPC, high GNL3 expression significantly correlated with reduced infiltration of multiple lymphoid and myeloid populations, including CD8+ T cells, NK cells, dendritic cells, and B cells (Figure S5E–I).

To investigate these observations further, we performed IMC analysis (Figure 6D) on the same PCa TMA used for GNL3 staining (Figure 1F–J). Cell populations were annotated based on a panel of 32 antibodies (Figure 6E,F). We found that tumor regions exhibited fewer CD8+ T cells compared to adjacent non‐tumor tissue (Figure 6G), and this reduction inversely correlated with elevated GNL3 expression (Figure 1I,J). In paired samples where tumor regions expressed higher GNL3 than adjacent non‐tumor regions, we detected diminished infiltration of CD8+ T cells, B cells, and T helper cells (Figure 6H; Figure S5J, K), suggesting that GNL3 contributes to CD8+ T cell elimination and promotes an immunosuppressive TME.

To corroborate this relationship, we analyzed a publicly available human PCa single‐cell RNA‐seq (scRNA‐seq) dataset [54]. Cell clusters were annotated into lymphoid and myeloid lineages (Figure 6I; Figure S5L), and patients were stratified based on GNL3 expression in tumor cells using pseudo‐bulking (Figure S5M). Tumors with high GNL3 expression exhibited a significantly reduced percentage of CD8+ T cells and NK cells (Figure 6J), reinforcing the link between GNL3 and elimination of cytotoxic immune cells.

**FIGURE 7:**
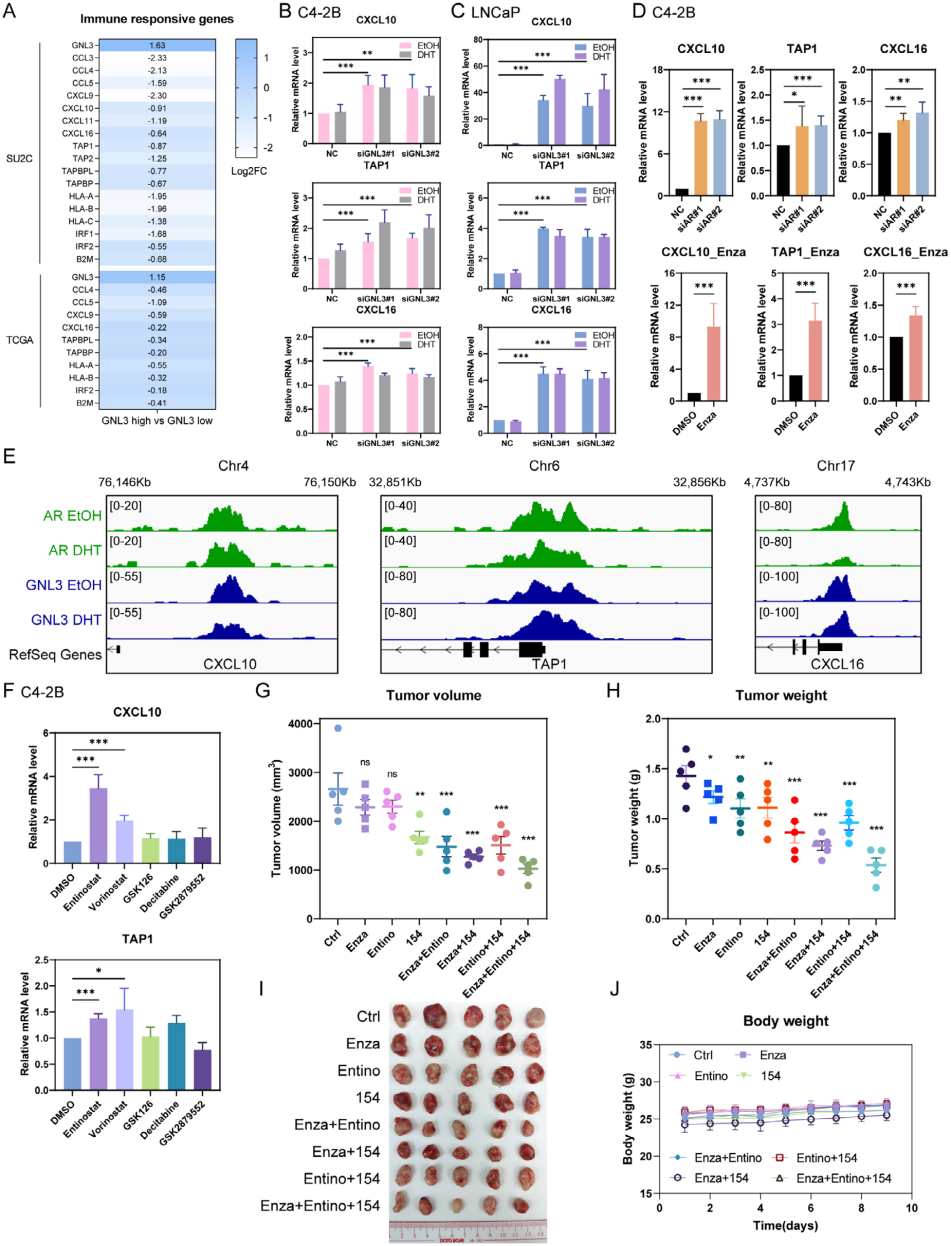
GNL3 diminishes CD8+ T cells infiltration and activation by repressing immune‐responsive genes CXCL10 and TAP1 (A) Heatmap showing expression of immune‐responsive genes in GNL3‐high vs. GNL3‐low PCa patients from SU2C and TCGA datasets. (B), (C) RT‐qPCR analysis of CXCL10, TAP1, and CXCL16 expression in C4‐2B (B) and LNCaP (C) cells following GNL3 knockdown. Error bars present ± SD (*n* = 3). *p*‐value: two‐way ANOVA. (D) RT‐qPCR analysis of CXCL10, TAP1, and CXCL16 expression in C4‐2B following AR knockdown and enzalutamide treatment. Error bars present ± SD (*n* = 3). *p*‐value: two‐way ANOVA. (E) Genomic tracks of AR and GNL3 binding peaks through ChIP‐seq analysis around CXCL10, TAP1, and CXCL16 in C4‐2B cells. (F) RT‐qPCR of CXCL10 and TAP1 expression in C4‐2B cells treated with epigenetic inhibitors. Error bars present ± SD (*n* = 3). *p*‐value: two‐way ANOVA. (G–J) In vivo efficacy of NEK2 inhibitor INH154, class I HDACs inhibitor Entinostat, and AR inhibitor enzalutamide in the RM1 mouse model. Tumor volume and weight are shown in G and H. Tumors were excised at the endpoint (I) (*n* = 5). Body weights are shown in J. Error bars present ± SEM. *p*‐value: two‐way ANOVA (compared with the control group). (^***^ *p* < 0.001; ^**^ *p* < 0.01; ^*^ *p* < 0.05; ns, not significant at the 0.05 level).

To investigate the underlying mechanism, we examined whether GNL3 represses immune‐responsive genes known to facilitate CD8+ T cell recruitment and activation, including CCL3, CCL4, CCL5, CXCL9, CXCL10, CXCL11, CXCL16, TAP1, TAP2, and HLA genes [55, 56]. A comparative gene expression analysis of PCa samples from the SU2C and TCGA datasets revealed lower expression of these genes in GNL3‐high tumors (Figure 7A). Moreover, GNL3 knockdown in both C4‐2B and LNCaP cells significantly elevated CXCL10, CXCL16, and TAP1 expression (Figure 7B,C; Figure S6A), indicating that GNL3 suppresses immune‐responsive gene expression.

To determine if AR also contributes to the repression of these genes, we examined their expression following AR knockdown or enzalutamide treatment. Both interventions significantly increased CXCL10 and TAP1 expression, with a modest increase in CXCL16 (Figure 7D; Figure S6B). Moreover, ChIP analysis revealed co‐binding of GNL3 and AR at the regulatory regions of these genes (Figure 7E; Figure S6C, D), supporting a model in which GNL3 and AR cooperatively suppress immune gene expression. Together, these findings uncover a novel immunosuppressive axis in PCa, wherein GNL3 and AR co‐repress immune‐responsive genes that promote CD8+ T cell infiltration and activation. This mechanism contributes to immune evasion and tumor progression, positioning GNL3 as a key regulator of the immunologically “cold” TME in advanced PCa.

### 2.7 Class I HDACs Mediate Epigenetic Repression of Immune‐Responsive Genes in PCa

To further dissect the mechanism by which GNL3 and AR transcriptionally co‐repress immune‐responsive genes such as CXCL10 and TAP1, we performed a targeted mini‐screen of epigenetic modifiers. Given the established role of epigenetic regulation in AR‐mediated transcriptional repression [12, 57, 58, 59, 60], we tested inhibitors of key epigenetic regulators, including the class I HDACs inhibitor Entinostat that targets HDAC1, HDAC2, and HDAC3, pan‐HDACs inhibitor Vorinostat, EZH2 inhibitor GSK126, DNMT inhibitor Decitabine, and LSD1 inhibitor GSK2879552. Among these inhibitors, Entinostat induced the expression of immune‐responsive genes CXCL10 and TAP1 most robustly (Figure 7F; Figure S6E), implicating class I HDACs as key epigenetic corepressors recruited by GNL3 and AR.

To further validate these findings, we treated cells with the highly selective HDAC1/2 inhibitor ACY‐957 [61]. Consistent with the Entinostat results, ACY‐957 treatment led to strong upregulation of both TAP1 and CXCL10 (Figure S6F), providing more selective pharmacological evidence that class I HDACs suppress these immune‐regulatory genes. These findings highlight HDAC inhibition as a potential strategy to reverse immunosuppression in PCa.

### 2.8 The Combination of NEK2, Class I HDACs, and AR Inhibitor Enhances Anti‐Tumor Efficiency In Vivo

To explore the therapeutic potential of co‐targeting NEK2, epigenetic regulators, and AR signaling in PCa, we assessed the in vivo efficacy of the NEK2 inhibitor INH154, class I HDACs inhibitor Entinostat, and the AR antagonist enzalutamide. Initial testing in the CRPC mouse cell line RM1 revealed a synergistic anti‐proliferative effect between INH154 and enzalutamide (Figure S6G). To investigate this synergy in an immune‐competent setting, RM1 cells were injected into C57BL/6 mice subcutaneously, followed by treatment with vehicle, single agents, dual combinations, or the triple‐drug regimen. Both dual and triple‐combination significantly reduced tumor volume and weight but not monotherapies (Figure 7G–I; Figure S6H), indicating enhanced anti‐tumor efficacy. Under the current dosing regimen and treatment condition, the triple therapy produced only a modest increase in tumor inhibition compared to the dual‐drug combination, suggesting further optimization of dosage and treatment duration will be required to fully assess therapeutic synergy. Importantly, no significant changes in body weight were observed across treatment arms (Figure 7J), suggesting that the combination was overall well tolerated.

Immunohistochemical analysis of treated tumors revealed increased CD8+ T cells and CXCL10 expression in both dual and triple‐combination groups, accompanied by a significant reduction in Ki67‐positive cells in the triple‐combination group (Figure S6I–K). Notably, CD8 and CXCL10 levels did not increase further with triple therapy relative to dual treatments under the current conditions, again highlighting the need for optimized treatment parameters to uncover the full synergistic potential of this regimen. Together, these findings demonstrate that simultaneously targeting NEK2, class I HDACs, and AR signaling elicits a potent anti‐tumor response in vivo. Moreover, this combinatorial strategy offers a promising therapeutic avenue for overcoming resistance and immune evasion in advanced PCa.

## 3 Discussion

AR signaling is a primary driver of PCa development and progression, orchestrating transcriptional programs through interacting with a diverse array of protein and non‐protein cofactors [7, 8, 9, 10, 11, 12]. Previous studies have implicated the oncogenic potential of AR coregulators such as p300, GRHL2, and TRIM33 [28, 62, 63], highlighting the importance of this regulatory axis. Herein, we employed a proteomics‐based method to comprehensively identify AR‐interacting proteins in primary PCa and CRPC cells. We discovered and validated GNL3 as a novel coregulator with dual functionality, acting as both a coactivator and corepressor.

GNL3 (also known as guanine nucleotide binding protein‐like 3 and nucleostemin) is predominantly expressed in stem and cancer cells [64, 65, 66, 67, 68, 69], where it binds with GTP and regulates cell cycle progression and apoptosis through a p53‐dependent mechanism [64, 70, 71]. GNL3 interacts with MDM2 to stabilize the protein and attenuate p53 transcription activity [72], and has been implicated in transcriptional regulation via long noncoding RNAs (lncRNAs) [73]. GNL3 modulates multiple oncogenic signaling pathways and is associated with the progression of multiple cancer types, including gastric, hepatocellular, colon, osteosarcoma, and lung cancers [65, 66, 67, 68, 69, 74]. Our findings extend these observations to PCa, demonstrating that GNL3 promotes tumor progression, metastatic potential, and immunosuppression in both primary PCa and CRPC.

Mechanistically, we show that GNL3 facilitates AR chromatin binding and coactivates transcription of genes involved in cell cycle and proliferation, such as NEK2 and CDC20. Simultaneously, GNL3 represses immune‐responsive genes, including CXCL10 and TAP1, contributing to CD8+ T cell exclusion and the establishment of an immunosuppressive TME. GNL3 expression increases progressively from normal prostate to CRPC and correlates with poor prognoses and increased risk of disease recurrence. Although our xenograft experiments validate a tumor‐promoting role for GNL3, the limited tumor take rate in castrated mice highlights the need for expanded cohorts to increase statistical power. Additional in vivo studies, including GNL3 overexpression in AR‐negative PCa models and AR knockdown combined with GNL3 perturbations, will be informative for delineating AR‐dependent and AR‐independent mechanisms of GNL3 action.

PCa is widely considered an immunologically “cold” tumor with limited responsiveness to immunotherapy [75, 76]. This phenotype is particularly pronounced in CRPC, which features a highly immunosuppressive microenvironment characterized by reduced CD8+ T cells and increased myeloid‐derived suppressor cells (MDSCs) [19, 77]. Although our deconvolution analysis showed no significant differences in monocyte or macrophage lineages between GNL3‐high and GNL3‐low patient groups, GNL3‐high patients exhibited significantly reduced dendritic cell and neutrophil signatures. Given the role of dendritic cells as potent antigen‐presenting cells (APC) essential for promoting the activation of anti‐tumor effect of CD8+ T cells [78, 79], their lower levels in GNL3‐high patients might further contribute to an immunosuppressive TME and cause resistance of therapies to PCa. These observations complement emerging evidence highlighting a central role for AR signaling in shaping this immune landscape. For instance, IL‐23 secreted by MDSCs activates AR signaling via the STAT3‐RORγ axis, promoting resistance to ADT [19]. Neutralization of IL‐23 restores sensitivity to ADT and enhances the efficacy of AR antagonists [19]. AR also directly represses IFNγ in CD8+ T cells, impairing their function and contributing to immunotherapy resistance [22]. AR inhibition restores IFNγ signaling and CD8+ T cell activation, improving responsiveness to immune checkpoint blockades [22]. More recently, AR was shown to transcriptionally repress MHCI expression in PCa cells, reducing antigen presentation and enabling immune evasion [23]. However, AR inhibition only transiently restores MHCI levels, suggesting the involvement of additional regulatory factors [23].

Our study builds upon these insights by identifying GNL3 as a novel AR coregulator that cooperatively represses immune‐responsive genes critical for CD8+ T cell recruitment and activation. Moreover, we showed that high GNL3 expression is associated with an immunosuppressive TME in human PCa. Although syngeneic mouse models would provide further in vivo support for the role of GNL3 in shaping the TME, our initial attempts to generate stable GNL3‐knockdown mouse PCa cell lines were unsuccessful due to insufficient knockdown efficiency. Thus, future optimization work will be required. Furthermore, transgenic mouse models with conditional modulation of GNL3 expression would offer a powerful system to further delineate the role of GNL3 in shaping the TME and regulating anti‐tumor immunity.

Through a targeted screen of epigenetic modifiers, we identified class I HDACs as key mediators of GNL3‐driven immune gene repression. Our preliminary data further suggest that HDAC1 plays an essential role in suppressing TAP1, as HDAC1 knockdown significantly increased TAP1 expression, whereas repression of CXCL10 may involve additional HDAC family members, warranting further investigation. These findings are strongly supported by prior studies demonstrating the role of HDACs in repressing immune responses in multiple cancer types [80, 81, 82]. For example, Sofia et al. [81]. showed that HDAC inhibitors such as Entinostat and Vorinostat sensitize prostate and breast cancer cells to CD8+ T cell‐mediated killing, and that siRNA‐mediated HDAC1 knockdown recapitulates this effect, identifying HDAC1 as a key mediator of immune evasion. Similarly, Hong et al. [82]. screened 97 compounds and found that only HDAC inhibitors robustly induced T‐cell attracting chemokines such as CXCL10, CXCL9, and CCL5, reinforcing the central role of HDACs in suppressing chemokine expression. Furthermore, Sean et al. [80]. demonstrated that HDAC inhibition enhances both antigen presentation (MHCI) and Cxcr3‐ligand (receptor of Cxcl10) signaling, promoting CD8+ T cell infiltration and overcoming resistance to immunotherapy in PCa. Their genetic models showed that HDAC1 knockdown transiently increased classical MHCI molecules (H2‐K^b^ and H2‐D^b)^, whereas overexpression suppressed them, further supporting our observations. Collectively, these results, together with extensive supporting literature, reinforce the conclusion that class I HDACs act downstream of the GNL3‐AR complex to suppress expression of CXCL10 and TAP1, thereby contributing to immune evasion in PCa.

While ADT remains the cornerstone of PCa treatment, resistance inevitably leads to CRPC [83]. Second‐generation AR antagonists such as enzalutamide and bicalutamide offer some survival benefits, but resistance to these agents remains a major clinical challenge [84, 85]. Although no GNL3‐specific inhibitors currently exist and GNL3 is categorized as pan‐essential in DepMap [86], our mechanistic analyses enabled the identification of downstream effectors, including NEK2 and class I HDACs, as tractable therapeutic targets. We showed that NEK2 inhibition synergized with AR blockade in vitro, and preliminary in vivo studies demonstrated potent anti‐tumor efficacy with dual and triple combinations. Future studies will systematically characterize the effect of these combinations on the immune cell populations within the TME.

In summary, we identified GNL3 as a novel AR coregulator with dual coactivator and corepressor functions in PCa. Our study uncovers a previously unrecognized mechanism by which the AR transcriptional complex integrates oncogenic signaling and immune suppression. Targeting GNL3, either directly or through rational combination therapies, represents a promising strategy to overcome therapeutic resistance and improve treatment outcomes in advanced PCa.

## 4 Materials and Methods

### 4.1 Cell Culture and Reagents

LNCaP (ATCC; CRL‐1740; RRID: CVCL_1379), 22Rv1(ATCC; CRL‐2505; RRID: CVCL_1045), and C4‐2B (ViroMed Laboratories) human PCa cell lines were purchased and used in this study. Mouse PCa cell line RM1 was kindly gifted by Professor Dingxiao Zhang (Hunan University). Early passages (< 20) of cells were used. The cells were confirmed mycoplasma‐free by the MycoAlert kit (Lonza). All cell lines were cultured in RPMI 1640 (Gibco) containing 1% penicillin‐streptomycin (Gibco) and 10% fetal bovine serum (FBS) (Gibco) in 37°C incubators with 5% CO_2_. 293T cells (gifted kindly from Professor Chuxia Deng, University of Macau) were cultured for a package of lentivirus using corresponding plasmids such as psPAX2, pMD2.G, and shGNL3 (OriGene, TL304295) plasmids. Lipofectamine 3000 reagents (Invitrogen, L3000015) were utilized to transfect plasmids. Dicer‐substrate short interfering RNAs (DsiRNAs) were obtained from IDT. Transient transfection of DsiRNAs was mediated by Lipofectamine RNAiMAX (Invitrogen, 13778150). For hormone deprivation of cellular experiments, cells were maintained in an androgen‐free medium that contained RPMI 1640 free of phenol red (Gibco) and 5% charcoal/dextran‐treated FBS (Cytiva, HyClone) for at least 72 h.

### 4.2 RNAi Studies, RNA Isolation, and Real‐Time qPCR

C4‐2B, 22Rv1, and LNCaP cells were transferred to 6‐well plates and maintained in androgen deprivation medium, followed by transfection of 10 nm DsiGNL3 using Lipofectamine RNAiMAX according to the manufacturer's protocol. Before collecting samples, cells were treated with 10 nm dihydrotestosterone (DHT). Control groups were treated with ethanol (EtOH) for the same period. For the experiments screening histone modifiers on immune‐responsive genes, cells were treated with multiple histone modifiers’ inhibitors, including Entinostat, Vorinostat, Decitabine, GSK126, and GSK2879552 (MedChemExpress, HY‐18632A). Entinostat, Vorinostat, and Decitabine were gifted generously by Professor Joong Sup Shim (University of Macau). GSK126 was gifted generously by Professor Gang Li (University of Macau). Next, collect samples and isolate RNA using the RNeasy Mini Kit (QIAGEN, 74106). A High‐Capacity cDNA Reverse Transcription Kit (Applied Biosystems, 4368813) was utilized to prepare cDNA as specified by the manufacturer. iTaq Universal SYBR Green Supermix (Bio‐Rad, 1725121) was used subsequently to quantify gene expression levels by quantitative real‐time PCR. The sequences of primers were provided in Table S4.

### 4.3 RNA‐seq and Data Analysis

Following extraction with TRIzol (Invitrogen, 15596026) as per the manufacturer's guidelines, total RNA was quantified and assessed for integrity using the Agilent 2100 Bioanalyzer in combination with RNA Nano 6000 Assay Kit. The sequencing library was constructed by the NEBNext Ultra II RNA Library Prep Kit for Illumina (NEB, E7770) according to the manufacturer's instructions. The NovaSeq 6000 Sequencing System was used for sequencing. The obtained raw reads were subjected to the FastQC package to assess the data quality. The alignment was performed using the HISAT2 [87] package against the human reference genome GRCh38 (hg38). FeatureCounts [88] was employed to quantify gene expression, and DESeq2 [89] was applied to identify transcripts showing differential expression.

### 4.4 Cell Proliferation Assay

C4‐2B, 22Rv1, and LNCaP cells were transferred to and cultured in 6‐well plates. The cells were transfected with DsiGNL3 or NC for 24 h. Then cells were collected and counted after trypsin digestion. A certain number of cells were transferred to 96‐well plates. AlamarBlue cell viability reagent (Invitrogen, DAL1100) was employed to examine cell viability daily as per the manufacturer's instructions. For the drug sensitivity test, a series of concentrations of enzalutamide (MedChemExpress, HY‐70002) and bicalutamide (MedChemExpress, HY‐14249) were added to 96‐well plates after cell seeding.

### 4.5 Colony Formation Assay

C4‐2B, 22Rv1, and LNCaP cells were transferred to and cultured in 6‐well plates. The cells were then transfected with DsiGNL3 or NC for 24 h. Following collection, cells were seeded at low density in a new 6‐well plate and cultured for a period of 2 or 3 weeks. The colony was formed and stained with crystal violet dye.

### 4.6 Drug Synergistic Assay

C4‐2B, LNCaP, and RM1 cells were transferred to 96‐well plates and cultured for 24 h. Then, treat cells with a series of dosages of enzalutamide or bicalutamide, combining with either INH154 (MedChemExpress, HY‐117154) or Apcin (MedChemExpress, HY‐110287). Cell viability was evaluated through AlamarBlue. HSA synergy score was calculated through a web tool, SynergyFinder [50].

### 4.7 Chromatin Immunoprecipitation (ChIP)‐seq and ChIP‐qPCR

ChIP‐qPCR was executed according to the methods described in our previous report [90]. In brief, culture 1 × 10^7^ C4‐2B or LNCaP cells in 15 cm dishes with hormone‐deprived medium for 72 h. Subsequently, cells were exposed to either EtOH or 100 nm DHT for 2 h before cross‐linking by 1% formaldehyde (Sigma, 252549). Cross‐linked cells were collected and immunoprecipitated with anti‐GNL3 (Santa Cruz, sc‐166460) or anti‐AR (Santa Cruz, sc‐13062) antibodies that were pre‐coated to protein G Dynabeads (Invitrogen, 10009D). DNA was subjected to overnight precipitation followed by de‐cross‐linking at 65°C. The products were subjected to qPCR or high‐throughput sequencing. Similarly, cells were transfected with 10 nm DsiGNL3 before performing AR ChIP‐qPCR in the GNL3 knockdown condition. Details of primers employed for the ChIP‐qPCR assay are provided in Table S4.

Paired‐end libraries of ChIP‐seq were sequenced on the Illumina NovaSeq PE150 platform. Raw sequencing reads were aligned with the hg38 reference genome by Bowtie v.0.12.9 [91] with default settings. Picard MarkDuplicates v.2.20 was used to mark potential PCR artifacts. Peak calling was executed through MACS v.1.4.2 [92] via a cut‐off of p‐value 1e‐04. To assess peak overlap across samples, the findOverlapsOfPeaks function from ChIPpeakAnno v.3.16.1 R package [93] was employed. Peak annotation was conducted using ChIPseeker v.1.18 [94]. The heatmap and tag density plots centered around the peak regions (±5 Kb) were generated using deepTools v.3.2.1 [95]. Motif enrichment was carried out through HOMER v4.11.1 [96] with default parameters.

### 4.8 RIME

AR‐RIME was conducted according to the description in the publication [27]. In brief, culture 2 × 10^7^ C4‐2B cells in 15 cm dishes with hormone‐deprived medium for 72 h. Then, cells were exposed to DHT (100 nm) for 2 h and cross‐linked with 1% methanol‐free formaldehyde (Thermo, 28908) at room temperature for 10 min. 10 µg anti‐AR (Santa Cruz, sc‐13062) or rabbit IgG (Santa Cruz, sc‐66931) antibody pre‐coated with protein G Dynabeads was applied for immunoprecipitation. Bound proteins on beads were extracted for 10 min in a denaturing buffer (0.02 m Tris‐HCl, pH 6.9, 2% SDS, 0.1 m dithiothreitol). The supernatant was harvested and frozen at −80°C for later proteomic analysis.

### 4.9 Label‐Free LC‐MS/MS Data Acquisition and Analysis

Protein digestions were performed through trypsin employing the filter‐aided sample preparation (FASP) protocol [97]. Next, purify the tryptic peptides using C18 ZipTips (Millipore, ZTC18S960) following the manufacturer's protocol. Following digestion and purification, the peptides were dried using a SpeedVac concentrator to remove residual solvents. The dried peptides were then carefully reconstituted in a solution containing 0.1% formic acid and 5% acetonitrile (ACN) to enhance solubility and ensure compatibility with LC‐MS/MS analysis. Peptide separation was carried out on an EASY‐Spray liquid chromatography column (75 µm × 50 cm, 2 µm, 100 Å pore size; Thermo, ES903). Then, elute the peptides over a 65‐min gradient ranging from 5% to 35% ACN in 0.1% formic acid, allowing for efficient resolution of complex peptide mixtures. The eluted peptides were subsequently analyzed in data‐dependent acquisition (DDA) mode and introduced into a Q Exactive mass spectrometer (Thermo). Acquire full MS scans over a mass‐to‐charge (m/z) range of 400–2,500 at a resolution of 70,000 (full width at half maximum, FWHM), ensuring high mass accuracy and sensitivity for peptide detection. The top 20 most intense precursor ions from each scan were automatically selected for fragmentation via higher‐energy collisional dissociation (HCD) with a normalized collision energy (NCE) of 25. Fragment ion (MS/MS) spectra were subsequently recorded at a resolution of 17,500 (FWHM), enabling precise identification of peptide sequences.

Data were processed using MaxQuant (v1.5.3.17) [98],  with database searching performed against the reviewed UniProt human protein database. Proteins were identified based on the detection of at least two unique peptides per protein. Stringent filtering criteria were applied, with peptide‐spectrum match (PSM), dependent peptide, and protein false discovery rates (FDRs) all controlled at 0.01. For quantitative analysis, label‐free quantification (LFQ) intensities were calculated using unique and razor peptides.

### 4.10 Coimmunoprecipitation (Co‐IP)

Co‐IP assays were carried out according to the RIME protocol with slight changes [27]. Briefly, 1 × 10^7^ C4‐2B or LNCaP cells were seeded in 15 cm dishes with a hormone‐deprived medium for 72 h. Nuclear lysates were prepared accordingly. Lysates were immunoprecipitated with anti‐GNL3 antibodies that were pre‐coated to protein G Dynabeads. Following immunoprecipitation, the bead‐bound protein complexes were finally eluted by boiling in the sample buffer of SDS‐PAGE at 95°C for 5 min to denature and release the bound proteins. The resulting samples were then subjected to Western blotting analysis to detect specific protein interactions. Primary antibodies used for Co‐IP and Western blotting included anti‐GNL3 (Santa Cruz, sc‐166460) and anti‐AR (Santa Cruz, sc‐816).

### 4.11 Proximity Ligation Assay (PLA)

PLA was carried out through a Sigma–Aldrich kit according to the manufacturer's illustration. C4‐2B or LNCaP cells were transferred to and deposited on glass slides in 6‐well plates, followed by exposure to either EtOH or 100 nm DHT for 2 h. Then fix the cells through 4% formaldehyde for 30 min and permeabilize the cells by 0.5% Triton X‐100 for 20 min following fixation. Then block the cells by incubation of Duolink Blocking Solution for 1 h at 37°C. Cells were exposed to anti‐GNL3 (Sigma, HPA036742) and anti‐AR (Agilent, M3562) antibodies overnight at 4°C following the blocking step. Cells were rinsed with 1× Wash Buffer A prior to incubation with Anti‐Rabbit PLUS (Sigma, DUO92002) and Anti‐Mouse MINUS (Sigma, DUO92004) PLA probe for 1 h at 37°C. Ligation of oligonucleotides and amplification to generate red fluorescence by Detection Reagents Red (Sigma, DUO92008). The Zeiss LSM710 confocal microscope was used for imaging. For PLA on PCa tissue, Detection Reagents Brightfield (Sigma, DUO92012) was utilized. The Zeiss Imager M2 microscope was used for imaging.

### 4.12 Immunohistochemistry (IHC) Analysis on Tissue Microarray (TMA)

Prostate tissue samples in TMA format, prepared as formalin‐fixed and paraffin‐embedded (FFPE), were sourced from Shanghai Outdo Biotech, including three normal prostate samples, 52 adjacent non‐tumor tissues, and 95 tumor samples with different Gleason scores (GS) (20 (GS = 6), 60 (GS = 7), 10 (GS = 8), and 5 (GS = 9). GNL3 IHC staining was performed on the TMA. The tissue section was first deparaffinized in xylene and rehydrated by a graded ethanol series. Then, Target Retrieval Solution (Agilent, Dako S2367, pH 9) was used for heat‐induced antigen retrieval. Following antigen retrieval, the TMA was incubated overnight at 4 °C with the primary antibody, anti‐GNL3 antibody (Sigma, HPA036742). Following thorough washing with 1× PBS, the TMA was incubated for 2 h with secondary antibody and visualized by the DAB Detection System (BOSTER). The slides were imaged by a Hamamatsu scanner (NanoZoomer S60). GNL3 IHC staining intensity was evaluated and categorized into four levels: negative, weak, moderate, and strong.

### 4.13 Imaging Mass Cytometry (IMC)

The IMC analysis was performed on the above‐mentioned TMA. A panel of 32 antibodies labeled with different lanthanide metals was used. Custom antibodies were conjugated through the Maxpar X8 kit according to specifications, followed by quantification, and stored as per the protocol provided by the manufacturer. The TMA slide underwent standard preparation, including baking, dewaxing, rehydration, and antigen retrieval, followed by blocking and overnight incubation with the antibody cocktail. Nuclear staining was performed using Iridium Intercalator. Image acquisition was conducted with daily instrument calibration, and laser ablation was performed at 1 µm resolution. Regions of interest (ROI) measuring 500 µm × 500 µm were selected in areas surrounding prostate glands for each patient. Raw IMC data were processed using Steinbock [99] for image conversion and segmentation via DeepCell's Mesmer model [100]. Single‐cell and marker expression data were analyzed. Scanpy [101] was used for cell phenotyping. Clustering was performed using PhenoGraph [102], and marker identification was conducted using the Wilcoxon rank‐sum test. Each cluster was manually interpreted and labeled based on the expected marker expression defined by the antibody panel.

### 4.14 Xenograft and Allograft Mouse Model

Experimental procedures involving a xenograft mouse model were performed as per institutional guidelines with approval from the Institutional Animal Care and Use Committee at the University of Macau. 293T cells were cultured for a package of lentivirus using corresponding plasmids, including psPAX2, pMD2.G, with shControl (shCtrl) or shGNL3 (OriGene, TL304295). C4‐2B cells were infected by the viral supernatant supplemented with 8 µg/ml polybrene for 24 h, followed by 2 µg/ml puromycin selection for 3 days. 2 × 10^6^ C4‐2B shCtrl or shGNL3 cells were injected subcutaneously into the dorsal flank of male nude mice or castrated nude mice. Continuous assessment of tumor volume was performed. Similarly, 1 × 10^6^ RM1 cells were implanted in male C57BL/6J mice. Once tumor volume reached approximately 50 mm^3^, the mice were randomly grouped into eight sets (*n*  =  5 per group). Mice were administered 10 mg/kg enzalutamide (MedChemExpress, HY‐70002), INH154 (MedChemExpress, HY‐117154), and Entinostat (MedChemExpress, HY‐12163) every other day by intraperitoneal injection starting on Day 1. Throughout the experiment, tumor size and mouse body weight were recorded each day. Determine the tumor volume via the formula V = ½ × (L × W^2^), in which W is the width, and L is the length of the tumor, measured using calipers.

### 4.15 Metastatic Mouse Model

Animal experiments procedures of the metastatic mouse model were conducted in full compliance with institutional and national ethical guidelines and technically as previously described [103]. Protocols in these animal experiments received approval by the Animal Care and Use Committee of the School of Basic Medical Sciences, Shanghai Medical College, Fudan University (Approval No. 20220228‐014). All procedures adhered to the guidelines outlined in the National Institutes of Health Guide for the Care and Use of Laboratory Animals. These protocols ensured that all animals received humane care under standardized and ethically compliant conditions.

For 22Rv1 cells used in the metastatic mouse model, lentiviral particles were produced using a third‐generation packaging system (pVSVG, pRSV‐Rev, and pMDLg/pRRE). 22Rv1‐Firefly luciferase IRES EGFP cells were infected with viral supernatant plus polybrene (8 µg/ml) and selected with puromycin (1 µg/ml) for 3 days before maintenance in puromycin‐containing medium.

To establish a spontaneous lung metastasis model, 2 × 10^6^ 22Rv1 shCtrl or shGNL3 PCa cells were resuspended in 1× PBS (100 µl) and injected intravenously via the tail vein into male NOD‐SCID mice aged six weeks. Tumor progression and pulmonary colonization were longitudinally monitored via bioluminescent imaging (BLI) using an in vivo imaging system (IVIS Spectrum). For signal acquisition, D‐Luciferin (Yeasen, Cat. 40901ES) was freshly prepared in PBS at 15 mg/ml (1 g in 66.7 ml) and administered intraperitoneally at 100 µl per mouse (1.5 mg/mouse). To verify successful tail vein injection, D‐Luciferin was immediately administered following cell infusion in both control and GNL3‐knockdown cohorts, and BLI imaging was promptly conducted. Weekly imaging commenced from week 6 post‐injection and continued thereafter to monitor metastatic burden dynamically. All procedures were executed following protocols approved by the Department of Laboratory Animal Science, Fudan University, ensuring scientific rigor and reproducibility.

### 4.16 Pseudotime Trajectory Analysis

To construct a cancer progression trajectory on the PCaProfiler atlas, we employed Slingshot (v2.10.0) [104] in the R statistical environment. Slingshot was an algorithm that allowed the determination of sample lineages and provided the advantage of identifying branching differentiation, if any. In brief, PCA dimensionality reduction was performed using the top 800 variable genes across all prostate specimens. Dimension‐reduced coordinates were subsequently used as input for Slingshot, followed by pseudotime inference on each prostate sample using default algorithm settings. The pseudotime score of each sample represents its progression status within the inferred trajectory.

### 4.17 Correlation of Pseudotime Score, Gene Expression, and Androgen Receptor Signaling

The AR activity score was calculated based on three AR signature gene lists [39, 40, 41] for each prostate specimen in the SU2C, TCGA‐PRAD, and UW‐TAN cohorts. Log2‐transformed FPKM expression was used as input for gene set variation analysis with the GSVA package (v1.50.5) [105] in R. The correlation between GNL3 expression and AR activity score in PCa samples was calculated using Pearson correlation. And the correlation between GNL3, NEK2, CDC20, immune response genes, and pseudotime score was calculated using Pearson correlation. Similarly, the correlation between GNL3 with the left genes was calculated using PCaProfiler atlas via Pearson correlation. To investigate downstream effectors of GNL3 and AR co‐activated genes, we overlapped correlation results with upregulated genes from GNL3 knockdown RNA‐seq results. To identify the biological processes associated with GNL3 expression, we used correlated genes as input to a hypergeometric test with the ClusterProfiler R package (v4.10.1) [106]. Here, we incorporated reference gene sets from Kyoto Encyclopedia of Genes and Genomes (KEGG), Gene Ontology (GO), Reactome Pathways (ReactomePA), and the Molecular Signature Database (MSigDb) for over‐representation analysis.

### 4.18 Immune Deconvolution Analysis of PCa

The tumor microenvironment composition of bulk RNA‐seq prostate specimens was estimated using the MCP‐counter R package (v1.2.0) [53]. Normal, primary PCa, and CRPC samples from the PCaProfiler atlas were categorized into GNL3 high (upper 90%) and low expression (lower 10%) subgroups as determined by batch‐corrected VST expression. MCP‐counter was used to calculate an abundance score for two stromal and eight immune populations, namely, endothelial cells, fibroblasts, CD8+ T cells, T lymphocytes, natural killer cells, B cells, cytotoxic lymphocytes, monocytes, neutrophils, and myeloid cells. The abundance scores were computed based on the highly specific gene expression profile of each cell type population. Statistical significance between the abundance scores of the GNL3 expression subgroups was evaluated through a two‐sided Student's *t*‐test.

### 4.19 Single‐Cell Analysis of PCa Immune Cell Composition

A published human PCa single‐cell RNA‐seq dataset [54] was used to study the impact of GNL3 on immune cell abundances. We checked the quality of the UMI matrix for each sample and removed cells with less than 200 genes (nFeature_RNA < 200), over 8,000 genes (nFeature_RNA > 8,000), or an abnormally high mitochondrial gene percentage (percent.mt > 20%). DoubleFinder (v2.0.4) [107] further screened the remaining cells to filter out potential doublet droplets. After quality filtering, 111,478 cells and 53,672 genes were retained. Post‐processed matrices were normalized by variance stabilization followed by Harmony integration on the first 30 principal components to remove technical covariates between samples. Linear patterns captured in the first 30 principal components were then used to create a UMAP embedding, which reveals the distinct cell populations. Clustering was performed at a resolution of 2.25 to distinguish the major cell populations, followed by manual annotation of these clusters using sets of well‐established marker genes for each cell type, according to the author's specification. To rank the prostate specimens based on GNL3 expression, we aggregated the read counts of PCa epithelial cells (luminal, basal, and club cells) for each sample, followed by counts per million log normalization. The top 5 PCa samples with the highest and lowest GNL3 expressions were defined as high‐ and low‐expression cohorts. The cell type composition within the TME of GNL3 high and low samples was determined by the proportion of each cell type within the total number of the sample. The two‐sided Student's *t*‐test was employed to determine statistical relevance.

### 4.20 Statistical Analysis

The detailed statistical analysis methods and sample size for each experiment were depicted in the corresponding figure legends. Statistical analysis methods included two‐sided Student's *t*‐test, two‐way ANOVA, Mann‐Whitney U test, Wilcoxon signed‐rank test, and Kruskal‐Wallis test. The Pearson correlation method was applied for correlation analysis. Kaplan‐Meier analysis and log‐rank test were used for survival analysis. Data analyses were performed using the R program and Excel. The data presented as mean ± SD or SEM with three replicates. Statistical significance was indicated when below 0.05. The following labels were presented for statistical significance: ^***^ *p* < 0.001; ^**^ *p* < 0.01; ^*^ *p* < 0.05; ns, not significant at the 0.05 level.

## Author Contributions

C.Z. and E.C. provided guidance and designed the project. C.Z., T.L.C., N.N., D.D., M.D., Z.D., W.L., K.M.K.L., G.‐H.W., T.C.W.P., C.‐X.D., and E.C.developed the methodology. C.Z., T.L.C., N.N., D.D., M.D., Z.D., W.L., and K.M.K.L.acquired the data. T.L.C., N.N., and Z.M. performed the bioinformatics analysis. C.Z., T.L.C., N.N., D.D., M.D., Z.M., Z.D., W.L., K.M.K.L., G.‐H. W., T.C.W.P., C.‐X. D., and E.C. analyzed and interpreted the data. C.Z., T.L. C., N.N., and E.C. wrote and revised the manuscript.

## Conflicts of Interest

The authors declare no conflicts of interest.

## Supporting information




**Supporting File 1**: advs74573‐sup‐0001‐SuppMat.pdf.

**Supporting File 2**: advs74573‐sup‐0002‐TableS1.xlsx.

**Supporting File 3**: advs74573‐sup‐0003‐TableS2.xlsx.

**Supporting File 4**: advs74573‐sup‐0004‐TableS3.xlsx.

**Supporting File 5**: advs74573‐sup‐0005‐TableS4.xlsx.

**Supporting File 6**: advs74573‐sup‐0006‐SuppData.zip.

## Data Availability

All sequencing datasets generated in this work are available in the NCBI GEO database under accession numbers GSE305109 and GSE305110. Proteomics data from mass spectrometry have been archived in the PRIDE database, part of the ProteomeXchange Consortium, under dataset ID PXD067138.
