# Supplementary material for: GNL3 Orchestrates AR Transcriptional Programs to Drive Castration‐Resistant Prostate Cancer and Immune Evasion
Source: Adv Sci (Weinh). 2026 Mar 2;13(26):e16411. doi: 10.1002/advs.202516411 (PMC13159112; doi:10.1002/advs.202516411)
Supplement: Supplementary file 1 — Supporting File 1: advs74573‐sup‐0001‐SuppMat.pdf. [file ADVS-13-e16411-s002.pdf]

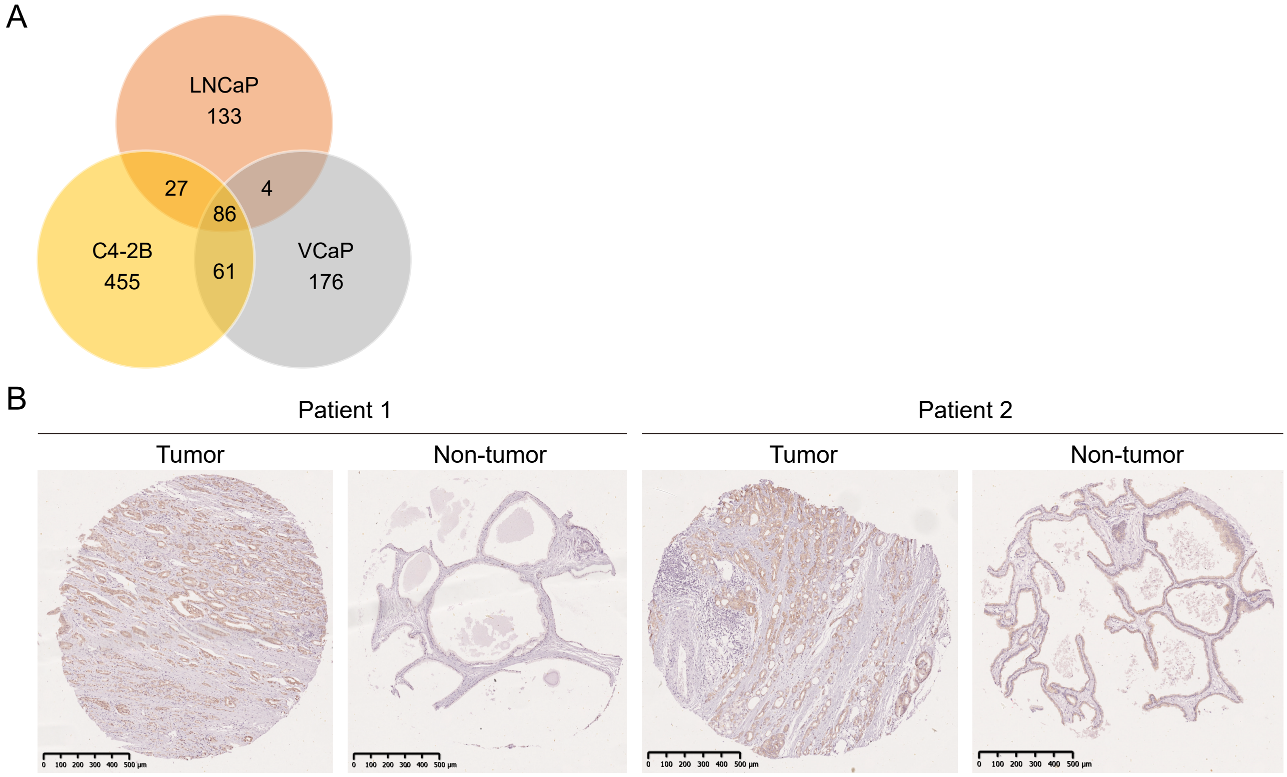

Supplementary Figure 1.
(A) Venn diagram representing the overlap of AR-associated proteins identified by RIME in C4-2B, LNCaP, and VCaP cells.
(B) Representative IHC images of GNL3 staining in tumor tissue and adjacent non-tumor tissue from a human PCa TMA. Scale bar: 500 μm.

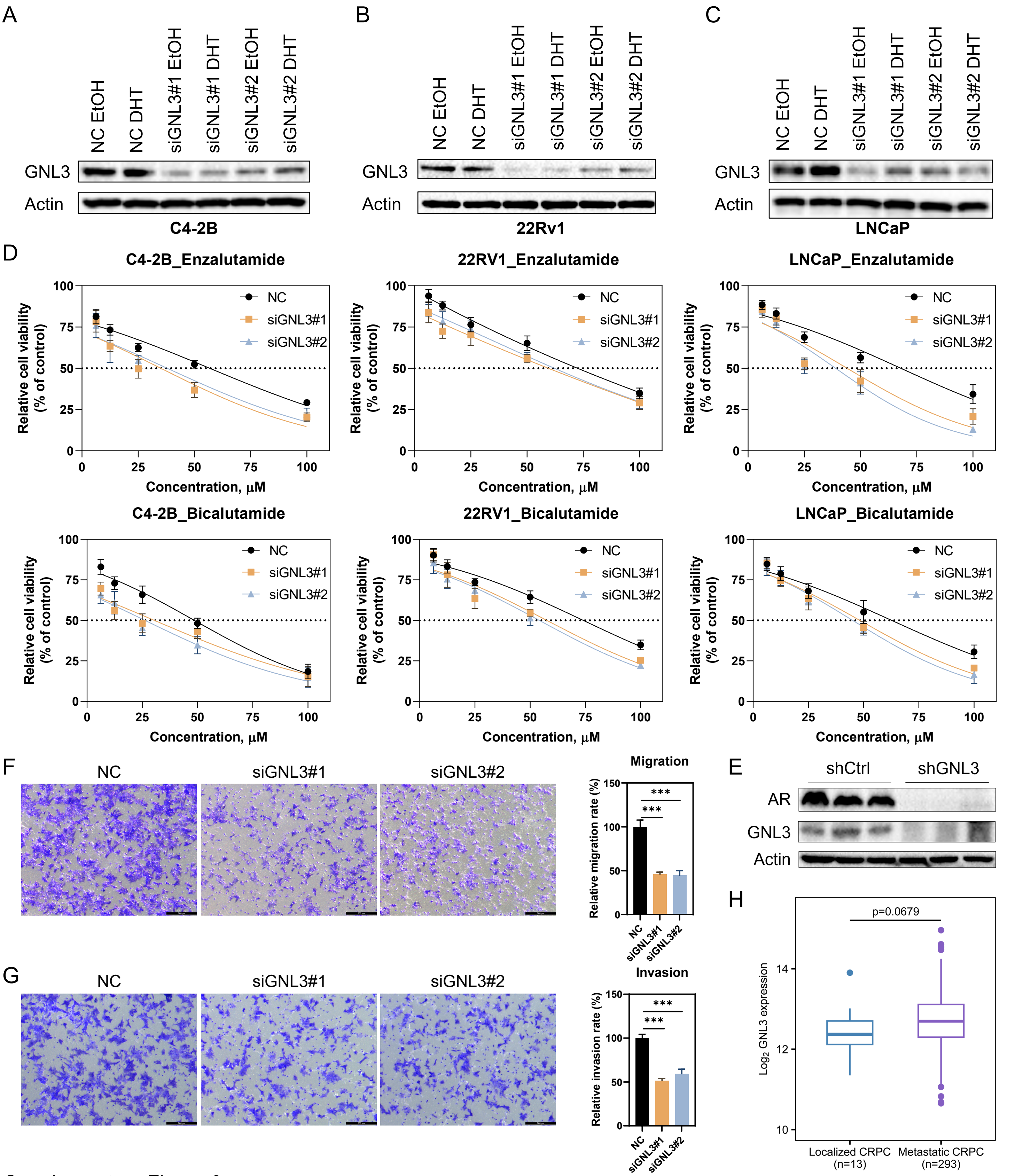

Supplementary Figure 2.
(A-C) GNL3 protein level analyzed by western blot in C4-2B, 22Rv1, and LNCaP cells after knockdown of GNL3 by siRNA.
(D) Dose-response curves for enzalutamide and bicalutamide in C4-2B, 22Rv1, and LNCaP with or without siGNL3.
(E) Western blot analysis showing GNL3 and AR protein level in tumors of Figure 2N.
(F-G) Migration (F) and invasion (G) of 22Rv1 cells after knockdown of GNL3. Scale bar: 200 μm. Error bars present ± SD (n = 3). P-value: two-way ANOVA.
(H) Boxplot comparing GNL3 expression in localized and metastatic CRPC samples in the PCaProfiler dataset. P-value: Mann-Whitney U test.
(***p < 0.001; **p < 0.01; *p < 0.05; ns, not significant at the 0.05 level)

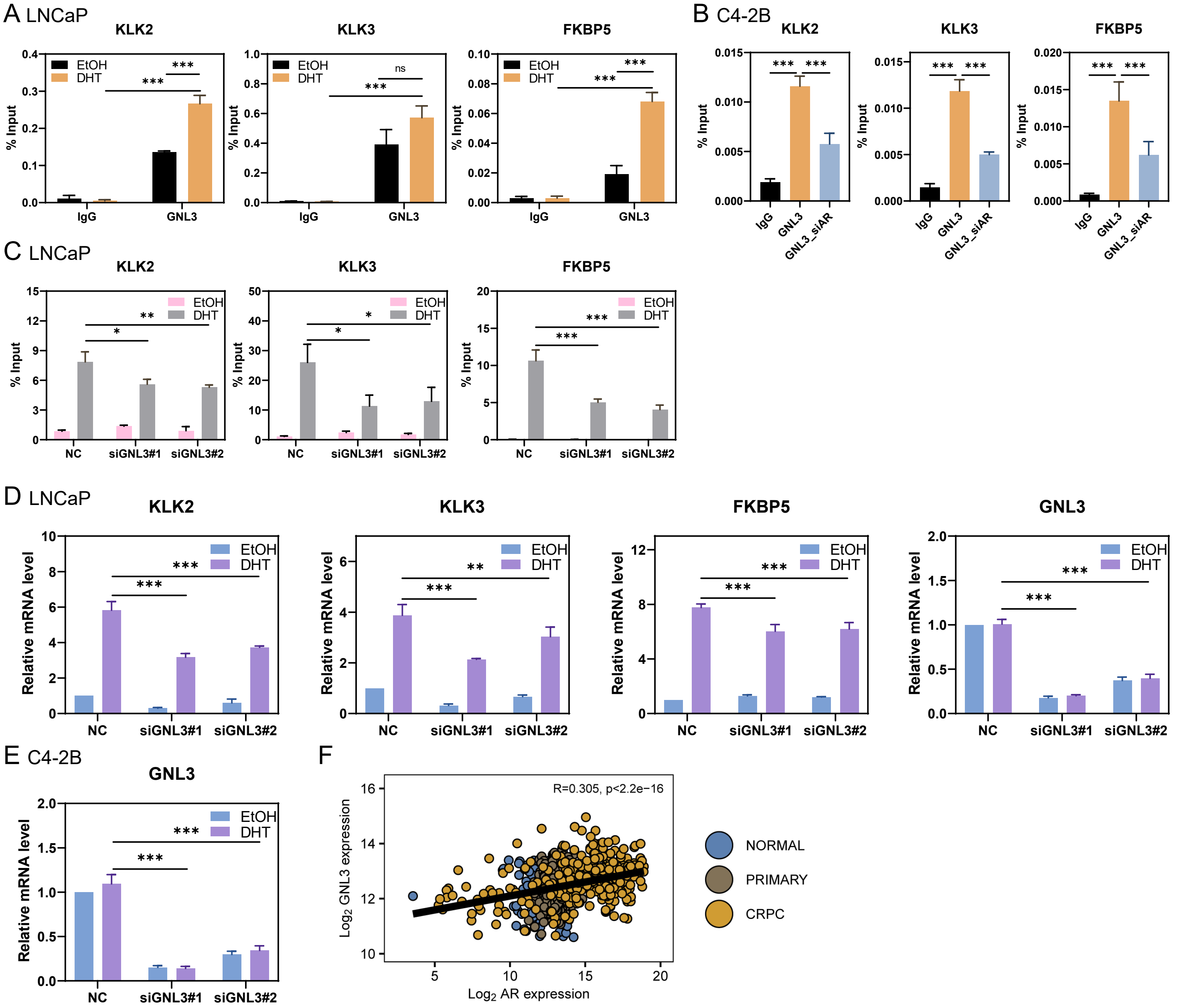

Supplementary Figure 3.

(A) GNL3 ChIP-qPCR at KLK2, KLK3, and FKBP5 genes in LNCaP cells with or without DHT treatment. The data are shown as a percentage of input. IgG was used for the negative control. Error bars present ± SEM (n = 3). P-value: two-way ANOVA.

(B) GNL3 ChIP-qPCR following AR knockdown at KLK2, KLK3, and FKBP5 genes in C4-2B cells. The data are shown as a percentage of input. Error bars present ± SD (n = 3). P-value: two-way ANOVA.

(C) AR ChIP-qPCR following GNL3 knockdown at KLK2, KLK3, and FKBP5 genes in LNCaP cells. The data are shown as a percentage of input. Error bars present ± SEM (n = 3). P-value: two-way ANOVA.

(D-E) RT-qPCR analysis of KLK2, KLK3, FKBP5 and GNL3 expression in LNCaP (D) and GNL3 expression in C4-2B (E) cells following GNL3 knockdown. Error bars present ± SD (n = 3). P-value: two-way ANOVA.

(F) Scatterplot showing the correlation between GNL3 and AR expression in the combined human prostate cancer dataset PCaProfiler (n=1,204). Pearson correlation and linear regression are shown.

(***p < 0.001; **p < 0.01; *p < 0.05; ns, not significant at the 0.05 level)

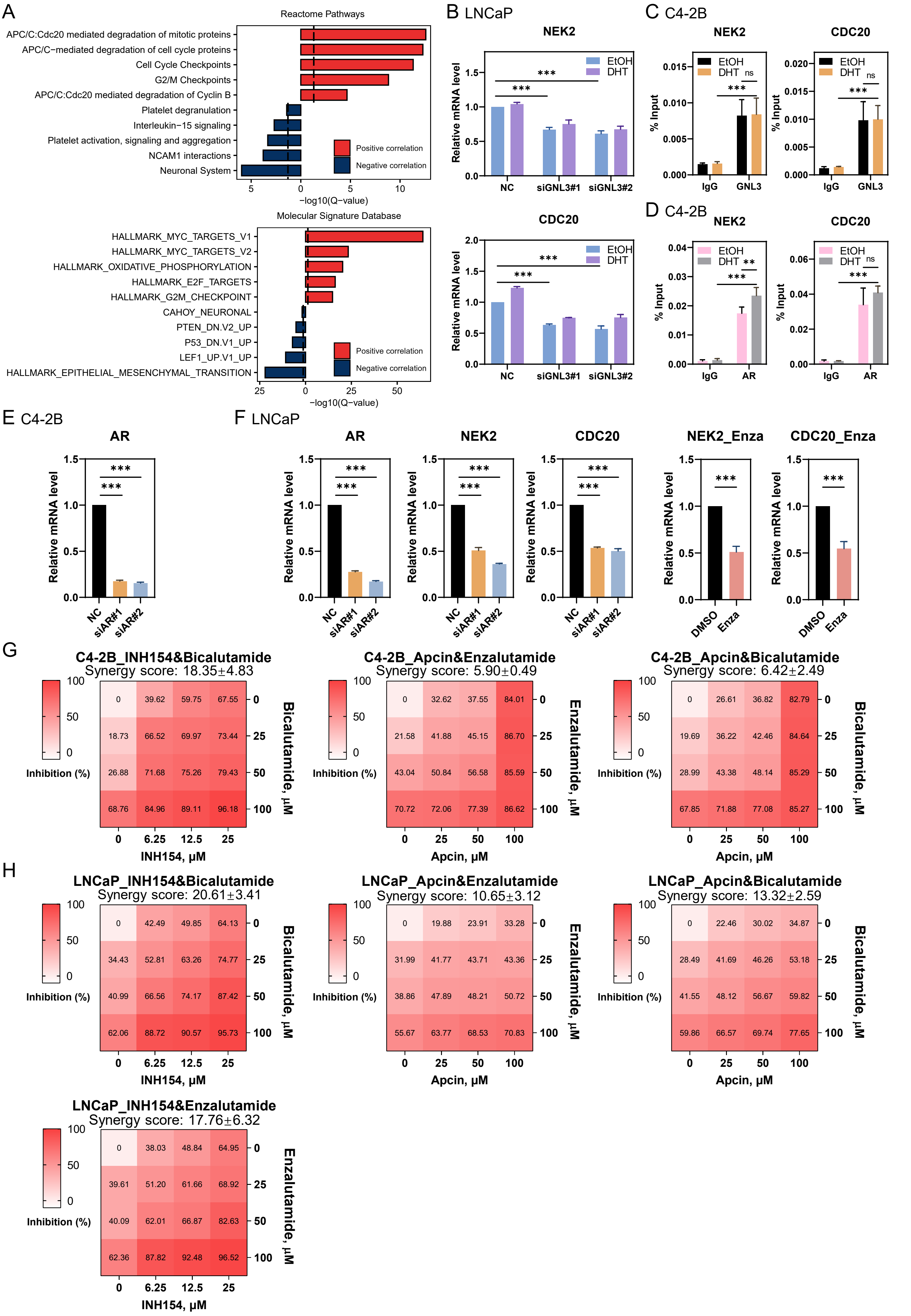

A
Reactome Pathways
APC/C:Cdc20 mediated degradation of mitotic proteins
APC/C−mediated degradation of cell cycle proteins
Cell Cycle Checkpoints
G2/M Checkpoints
APC/C:Cdc20 mediated degradation of Cyclin B
Platelet degranulation
Interleukin−15 signaling
Platelet activation, signaling and aggregation
NCAM1 interactions
Neuronal System
Positive correlation
Negative correlation
5 0 5 10
−log10(Q−value)
Molecular Signature Database
HALLMARK_MYC_TARGETS_V1
HALLMARK_MYC_TARGETS_V2
HALLMARK_OXIDATIVE_PHOSPHORYLATION
HALLMARK_E2F_TARGETS
HALLMARK_G2M_CHECKPOINT
CAHOY_NEURONAL
PTEN_DN.V2_UP
P53_DN.V1_UP
LEF1_UP.V1_UP
HALLMARK_EPITHELIAL_MESENCHYMAL_TRANSITION
Positive correlation
Negative correlation
25 0 25 50
−log10(Q−value)
B LNCaP
NEK2
CDC20
Relative mRNA level
EtOH
DHT
***
NC siGNL3#1 siGNL3#2
C C4-2B
NEK2
CDC20
% Input
EtOH
DHT
ns
***
IgG GNL3
D C4-2B
NEK2
CDC20
% Input
EtOH
DHT
**
ns
***
IgG AR
E C4-2B
AR
Relative mRNA level
***
NC siAR#1 siAR#2
F LNCaP
AR
NEK2
CDC20
NEK2_Enza
CDC20_Enza
Relative mRNA level
***
NC siAR#1 siAR#2
DMSO Enza
G
C4-2B_INH154&Bicalutamide
Synergy score: 18.35±4.83
Inhibition (%)
Bicalutamide, μM
INH154, μM
0 39.62 59.75 67.55
18.73 66.52 69.97 73.44
26.88 71.68 75.26 79.43
68.76 84.96 89.11 96.18
C4-2B_Apcin&Enzalutamide
Synergy score: 5.90±0.49
Enzalutamide, μM
Apcin, μM
0 32.62 37.55 84.01
21.58 41.88 45.15 86.70
43.04 50.84 56.58 85.59
70.72 72.06 77.39 86.62
C4-2B_Apcin&Bicalutamide
Synergy score: 6.42±2.49
Bicalutamide, μM
Apcin, μM
0 26.61 36.82 82.79
19.69 36.22 42.46 84.64
28.99 43.38 48.14 85.29
67.85 71.88 77.08 85.27
H
LNCaP_INH154&Bicalutamide
Synergy score: 20.61±3.41
Bicalutamide, μM
INH154, μM
0 42.49 49.85 64.13
34.43 52.81 63.26 74.77
40.99 66.56 74.17 87.42
62.06 88.72 90.57 95.73
LNCaP_Apcin&Enzalutamide
Synergy score: 10.65±3.12
Enzalutamide, μM
Apcin, μM
0 19.88 23.91 33.28
31.99 41.77 43.71 43.36
38.86 47.89 48.21 50.72
55.67 63.77 68.53 70.83
LNCaP_Apcin&Bicalutamide
Synergy score: 13.32±2.59
Bicalutamide, μM
Apcin, μM
0 22.46 30.02 34.87
28.49 41.69 46.26 53.18
41.55 48.12 56.67 59.82
59.86 66.57 69.74 77.65
LNCaP_INH154&Enzalutamide
Synergy score: 17.76±6.32
Enzalutamide, μM
INH154, μM
0 38.03 48.84 64.95
39.61 51.20 61.66 68.92
40.09 62.01 66.87 82.63
62.36 87.82 92.48 96.52

Supplementary Figure 4.
(A) Reactome and Hallmark pathway enrichment of GNL3 positively and negatively correlated genes in Figure 5B.
(B) RT-qPCR analysis of NEK2 and CDC20 expression in LNCaP cells following GNL3 knockdown. Error bars present ± SD (n = 3). P-value: two-way ANOVA.
(C-D) GNL3 (C) and AR (D) ChIP-qPCR at NEK2 and CDC20 in C4-2B cells with or without DHT treatment. Data are shown as a percentage of input. IgG was used for the negative control. Error bars present ± SD (n = 3). P-value: two-way ANOVA.
(E) RT-qPCR analysis of AR expression following AR knockdown in C4-2B cells. Error bars present ± SD (n = 3). P-value: two-way ANOVA.
(F) RT-qPCR analysis of NEK2 and CDC20 expression following AR knockdown or enzalutamide treatment in LNCaP cells. Error bars present ± SD (n = 3). P-value: two-way ANOVA.
(G-H) Heatmap showing cell inhibition percentages and synergy scores for drug combination (INH154 or Apcin with enzalutamide or bicalutamide) in C4-2B (G) and LNCaP (H) cells.
(***p < 0.001; **p < 0.01; *p < 0.05; ns, not significant at the 0.05 level)

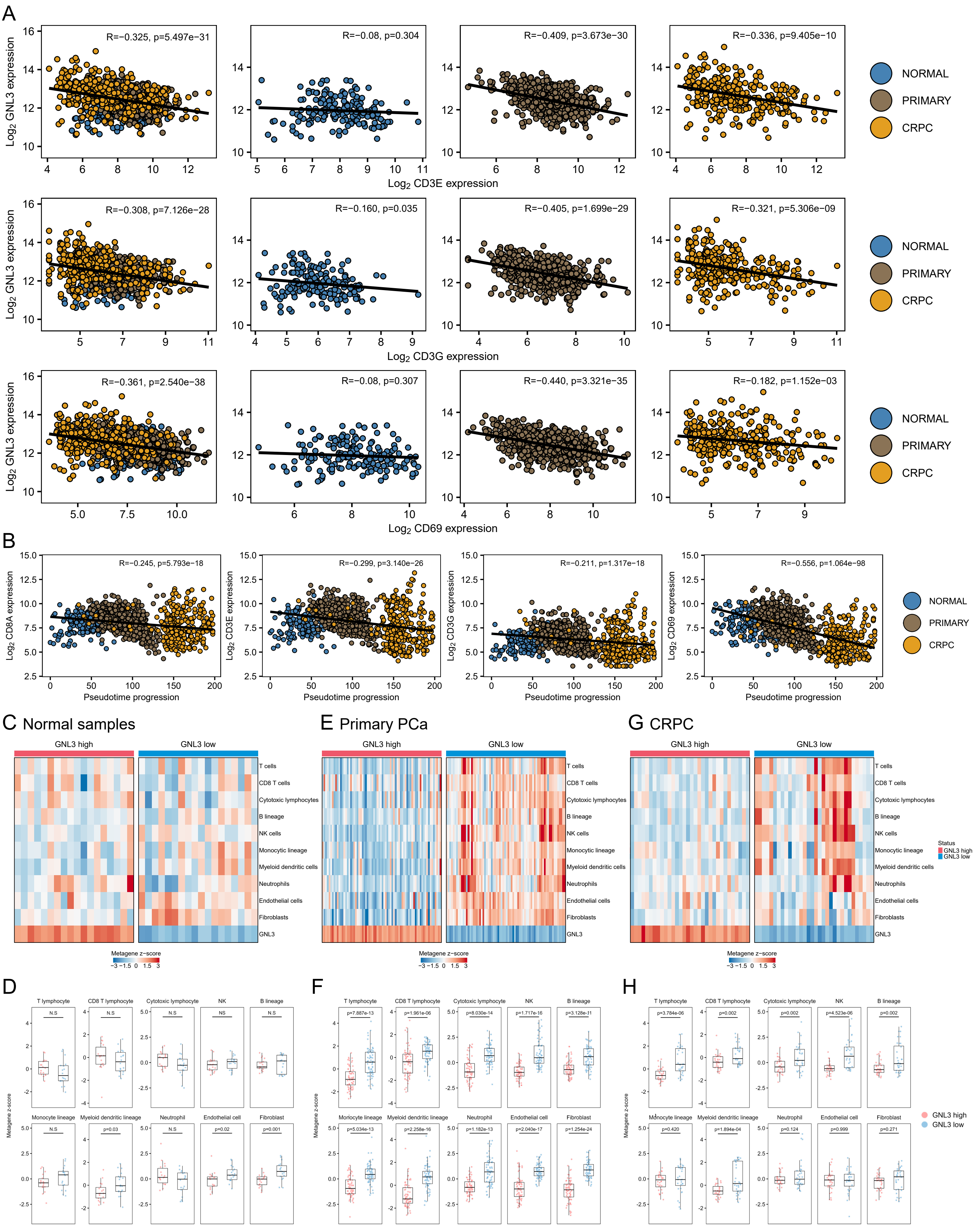
A
R=−0.325, p=5.497e−31
R=−0.08, p=0.304
R=−0.409, p=3.673e−30
R=−0.336, p=9.405e−10
NORMAL
PRIMARY
CRPC
Log2 GNL3 expression
Log2 CD3E expression
R=−0.308, p=7.126e−28
R=−0.160, p=0.035
R=−0.405, p=1.699e−29
R=−0.321, p=5.306e−09
Log2 CD3G expression
R=−0.361, p=2.540e−38
R=−0.08, p=0.307
R=−0.440, p=3.321e−35
R=−0.182, p=1.152e−03
Log2 CD69 expression
B
R=−0.245, p=5.793e−18
R=−0.299, p=3.140e−26
R=−0.211, p=1.317e−18
R=−0.556, p=1.064e−98
Log2 CD8A expression
Log2 CD3E expression
Log2 CD3G expression
Log2 CD69 expression
Pseudotime progression
C Normal samples
E Primary PCa
G CRPC
GNL3 high
GNL3 low
T cells
CD8 T cells
Cytotoxic lymphocytes
B lineage
NK cells
Monocytic lineage
Myeloid dendritic cells
Neutrophils
Endothelial cells
Fibroblasts
GNL3
Metagene z-score
Status
GNL3 high
GNL3 low
D
F
H
T lymphocyte
CD8 T lymphocyte
Cytotoxic lymphocyte
NK
B lineage
Monocyte lineage
Myeloid dendritic lineage
Neutrophil
Endothelial cell
Fibroblast
Metagene z-score
N.S
NS
p=0.03
p=0.02
p=0.001
p=7.887e-13
p=1.961e-06
p=8.030e-14
p=1.717e-16
p=3.128e-11
p=5.034e-13
p=2.258e-16
p=1.182e-13
p=2.040e-17
p=1.254e-24
p=3.784e-06
p=0.002
p=4.523e-06
p=0.420
p=1.894e-04
p=0.124
p=0.999
p=0.271
GNL3 high
GNL3 low

I

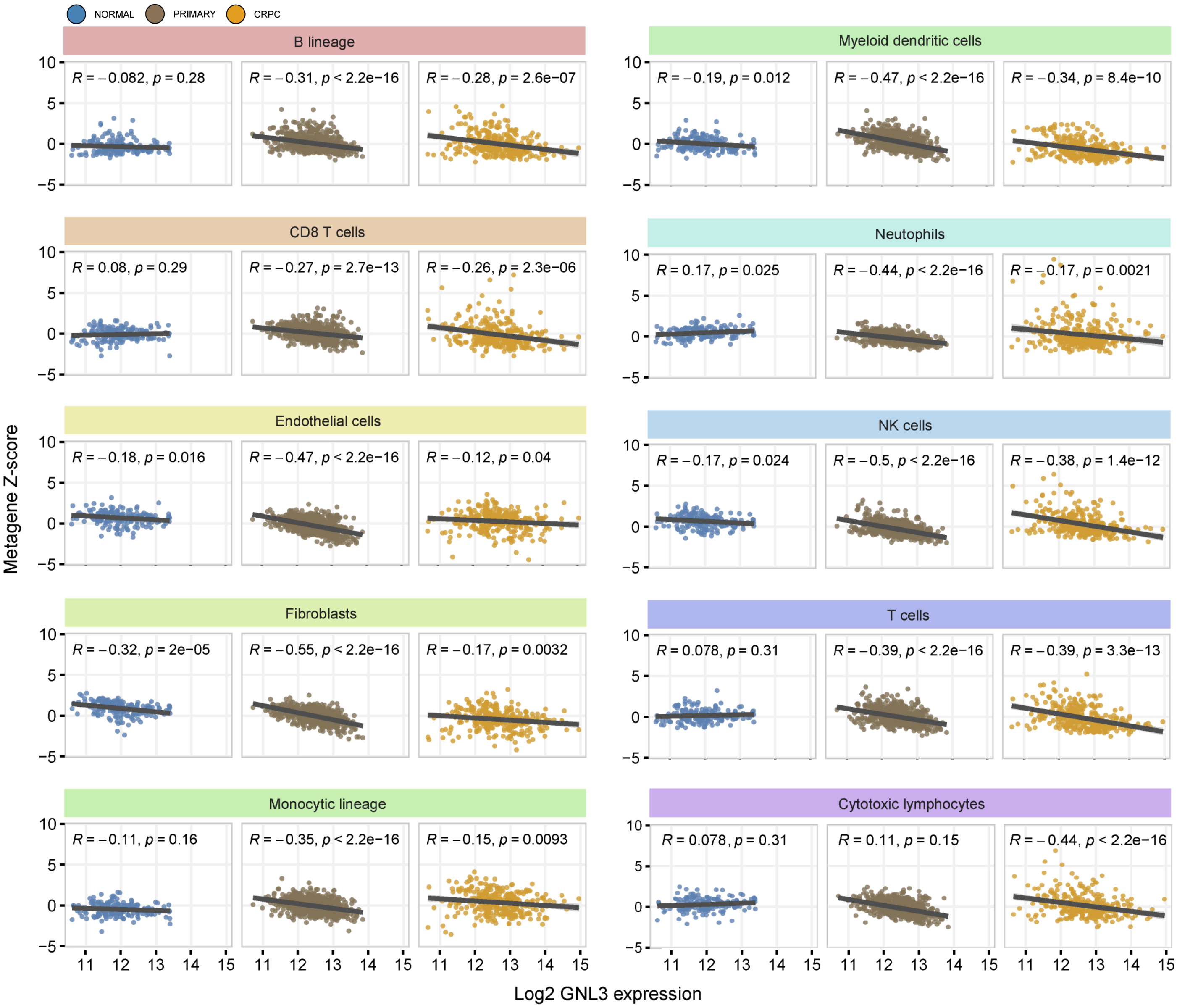
NORMAL
PRIMARY
CRPC
B lineage
R = −0.082, p = 0.28
R = −0.31, p < 2.2e−16
R = −0.28, p = 2.6e−07
Myeloid dendritic cells
R = −0.19, p = 0.012
R = −0.47, p < 2.2e−16
R = −0.34, p = 8.4e−10
CD8 T cells
R = 0.08, p = 0.29
R = −0.27, p = 2.7e−13
R = −0.26, p = 2.3e−06
Neutophils
R = 0.17, p = 0.025
R = −0.44, p < 2.2e−16
R = −0.17, p = 0.0021
Endothelial cells
R = −0.18, p = 0.016
R = −0.47, p < 2.2e−16
R = −0.12, p = 0.04
NK cells
R = −0.17, p = 0.024
R = −0.5, p < 2.2e−16
R = −0.38, p = 1.4e−12
Fibroblasts
R = −0.32, p = 2e−05
R = −0.55, p < 2.2e−16
R = −0.17, p = 0.0032
T cells
R = 0.078, p = 0.31
R = −0.39, p < 2.2e−16
R = −0.39, p = 3.3e−13
Monocytic lineage
R = −0.11, p = 0.16
R = −0.35, p < 2.2e−16
R = −0.15, p = 0.0093
Cytotoxic lymphocytes
R = 0.078, p = 0.31
R = 0.11, p = 0.15
R = −0.44, p < 2.2e−16
Metagene Z-score
Log2 GNL3 expression

J

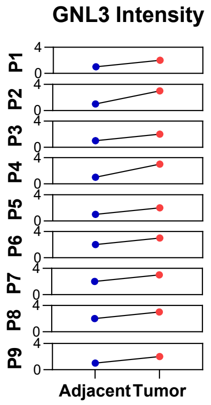
GNL3 Intensity
P1
P2
P3
P4
P5
P6
P7
P8
P9
Adjacent
Tumor

K

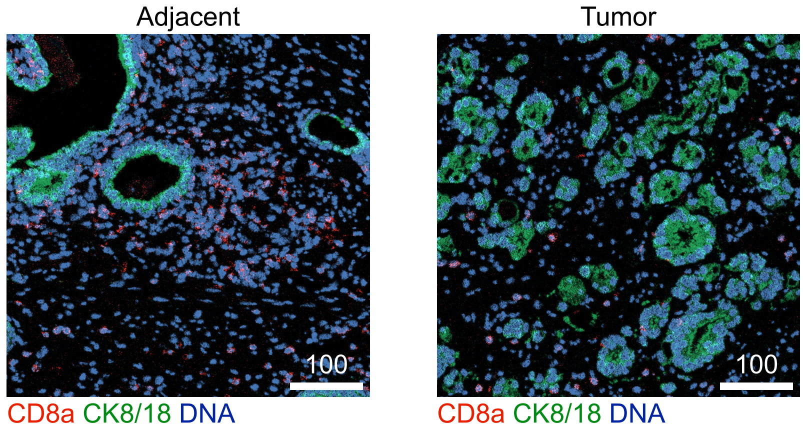
Adjacent
Tumor
100
100
CD8a CK8/18 DNA
CD8a CK8/18 DNA

L

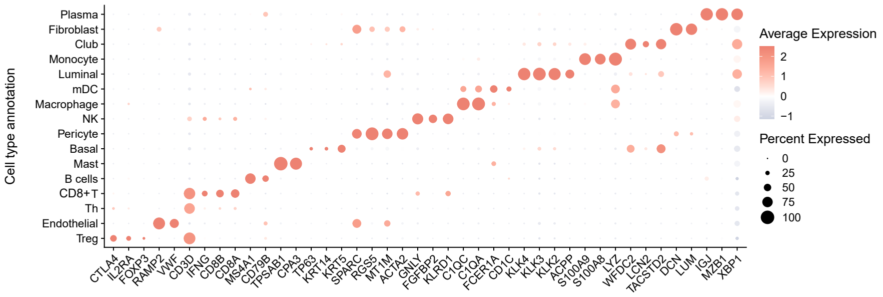
Cell type annotation
Plasma
Fibroblast
Club
Monocyte
Luminal
mDC
Macrophage
NK
Pericyte
Basal
Mast
B cells
CD8+T
Th
Endothelial
Treg
CTLA4
IL2RA
FOXP3
RAMP2
VWF
CD3D
IFNG
CD8B
CD8A
MS4A1
CD79B
TPSAB1
CPA3
TP63
KRT14
KRT5
SPARC
RGS5
MT1M
ACTA2
GNLY
FGFBP2
KLRD1
C1QC
C1QA
FCER1A
CD1C
KLK4
KLK3
KLK2
ACPP
S100A9
S100A8
LYZ
WFDC2
LCN2
TACSTD2
DCN
LUM
IGJ
MZB1
XBP1
Average Expression
Percent Expressed

M

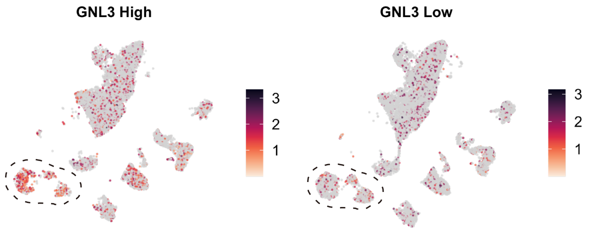
GNL3 High
GNL3 Low

Supplementary Figure 5.
(A) Scatterplot showing correlation between GNL3 and CD3E, CD3G, or CD69 expression across all samples and stratified by normal, primary PCa only, and CRPC only in the PCaProfiler dataset. Pearson correlation coefficient was calculated, and the associated p-value was used.
(B) Scatterplot showing correlation between pseudotime score and CD8A, CD3E, CD3G, or CD69 expression in the PCaProfiler dataset. Correlation is modelled by linear regression. Pearson correlation coefficient was calculated, and the associated p-value was used.
(C-H) MCP-counter immune deconvolution analysis of normal (C-D), primary PCa (E-F) and CRPC (G-H) samples stratified by GNL3 expression. MCP-counter abundance score was transformed through z-score. P-value: two-sided Student's t-test.
(I) MCP-counter immune deconvolution analysis illustrating correlations of immune cell populations with GNL3 expression in the PCaProfiler dataset. The MCP-counter abundance score was transformed through z-score. Pearson correlation coefficient was calculated, and the associated p-value was used.
(J) Paired dot plots visualizing the GNL3 intensity for patients in Figure 6H. (1-weak; 2-moderate; 3-strong.)
(K) Representative IMC images of CD8 staining in the human PCa tissue microarray. Scale bar: 100 μm.
(L) Dotplot showing expression of cell-type specific markers utilized for cluster annotation in Figure 6I. The color represents the expression level of a marker, while size indicates the percentage of cells expressing it.
(M) UMAP visualization of tumor cells for pseudo-bulking of GNL3-high and GNL3-low PCa patients in single-cell RNA-seq data from Figure 6J.

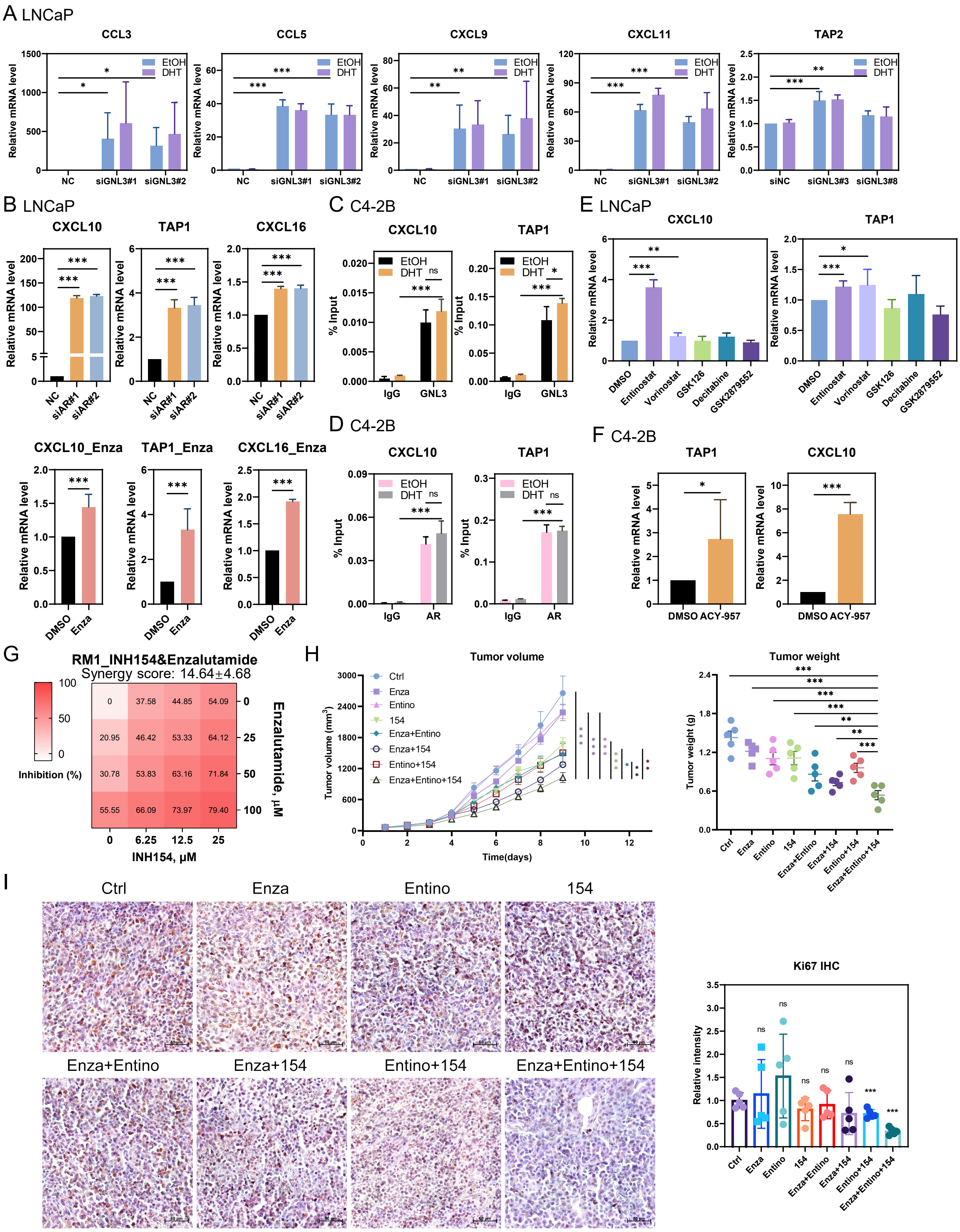

A LNCaP
CCL3
CCL5
CXCL9
CXCL11
TAP2
Relative mRNA level
EtOH
DHT
NC
siGNL3#1
siGNL3#2
siNC
siGNL3#3
siGNL3#8
B LNCaP
CXCL10
TAP1
CXCL16
siAR#1
siAR#2
CXCL10_Enza
TAP1_Enza
CXCL16_Enza
DMSO
Enza
C C4-2B
% Input
IgG
GNL3
D C4-2B
AR
E LNCaP
Entinostat
Vorinostat
GSK126
Decitabine
GSK2879552
F C4-2B
ACY-957
G
RM1_INH154&Enzalutamide
Synergy score: 14.64±4.68
Inhibition (%)
INH154, μM
Enzalutamide, μM
H
Tumor volume
Tumor volume (mm3)
Time(days)
Ctrl
Enza
Entino
154
Enza+Entino
Enza+154
Entino+154
Enza+Entino+154
Tumor weight
Tumor weight (g)
I
Ki67 IHC
Relative intensity

J

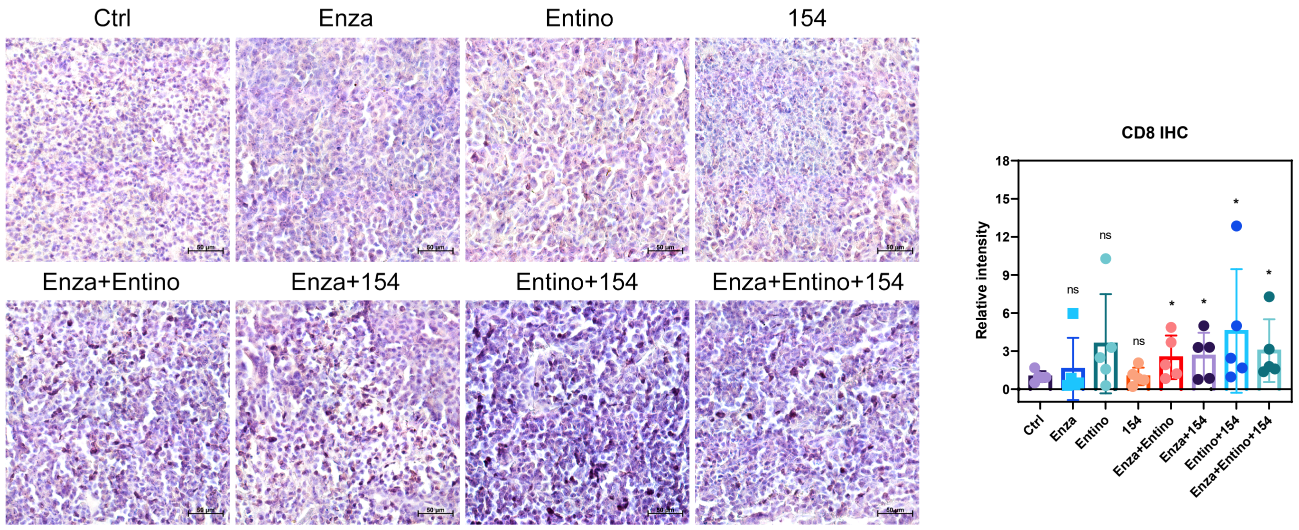

K

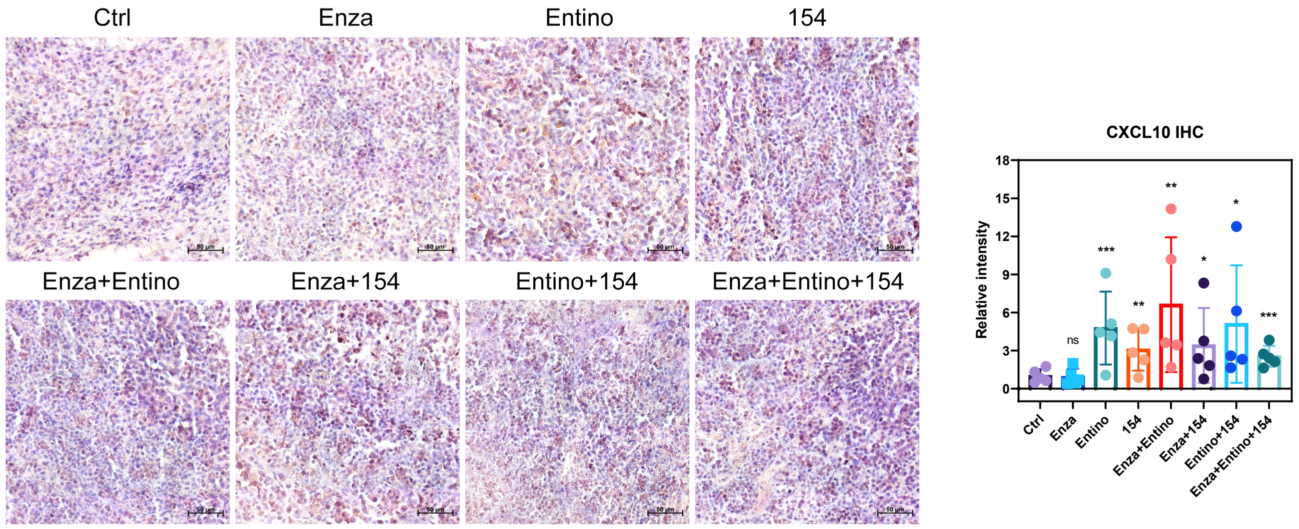

Supplementary Figure 6.
(A) RT-qPCR analysis of CCL3, CCL5 CXCL9, CXCL11, and TAP2 in LNCaP cells following GNL3 knockdown. Error bars present ± SD (n = 3). P-value: two-way ANOVA.
(B) RT-qPCR analysis of CXCL10, TAP1, and CXCL16 in LNCaP cells following AR knockdown and enzalutamide treatment. Error bars present ± SD (n = 3). P-value: two-way ANOVA.
(C-D) GNL3 (C) and AR (D) ChIP-qPCR at CXCL10 and TAP1 in C4-2B cells with or without DHT treatment. Data are shown as a percentage of input. IgG was used for the negative control. Error bars present ± SD (n = 3). P-value: two-way ANOVA.
(E) RT-qPCR analysis of CXCL10 and TAP1 in LNCaP cells treated with histone modifier inhibitors. Error bars present ± SD (n = 3). P-value: two-way ANOVA.
(F) RT-qPCR analysis of CXCL10 and TAP1 in C4-2B cells treated with ACY-957. Error bars present ± SD (n = 3). P-value: two-way ANOVA.
(G) Heatmap showing cell inhibition and synergy scores for INH154 with enzalutamide in RM1 cells.
(H) Tumor volume and tumor weight in Figure 7G-H. Error bars present ± SEM. P-value: two-way ANOVA (compared with triple-drug combination group).
(I-K) Representative IHC images of Ki67(I), CD8(J) and CXCL10(K) staining of tumor tissue in Figure 7I. Scale bar: 50 μm. Error bars present ± SD. P-value: two-way ANOVA (compared with control group).
(***p < 0.001; **p < 0.01; *p < 0.05; ns, not significant at the 0.05 level)

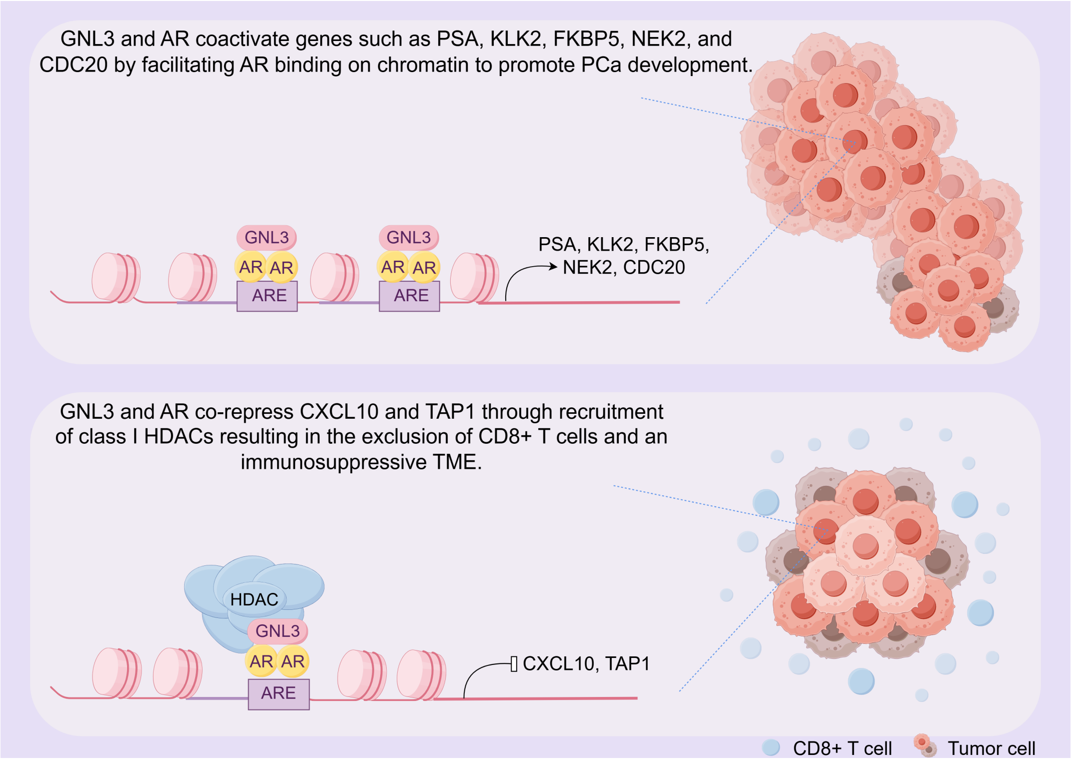

Abstract graph

A schematic illustration of GNL3 and AR regulation of gene transcription in PCa. GNL3 is highly expressed in PCa and functions as a dual AR regulator. Together with AR, GNL3 coactivates genes such as PSA, KLK2, FKBP5, NEK2, and CDC20, promoting tumor growth. Concurrently, GNL3 and AR co-repress immune-responsive genes, including CXCL10 and TAP1, via recruitment of class I HDACs, leading to the exclusion of CD8+ T cells and the establishment of an immunosuppressive TME in PCa. The graph was generated through Figdraw (https://www.figdraw.com/#/).
