# Supplementary figures and images for: GNL3 Orchestrates AR Transcriptional Programs to Drive Castration‐Resistant Prostate Cancer and Immune Evasion
Source: Adv Sci (Weinh). 2026 Mar 2;13(26):e16411. doi: 10.1002/advs.202516411 (PMC13159112; doi:10.1002/advs.202516411)

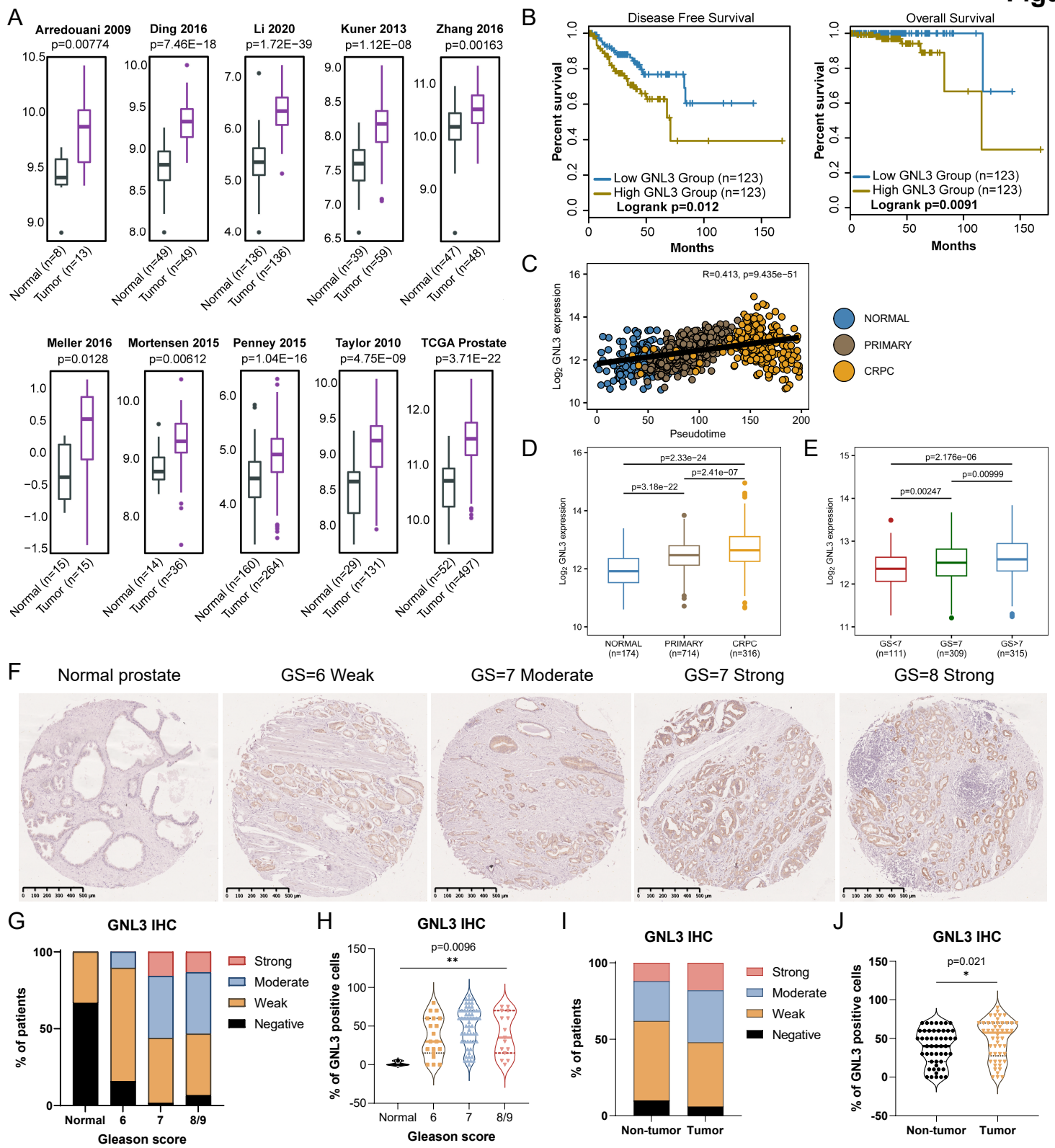

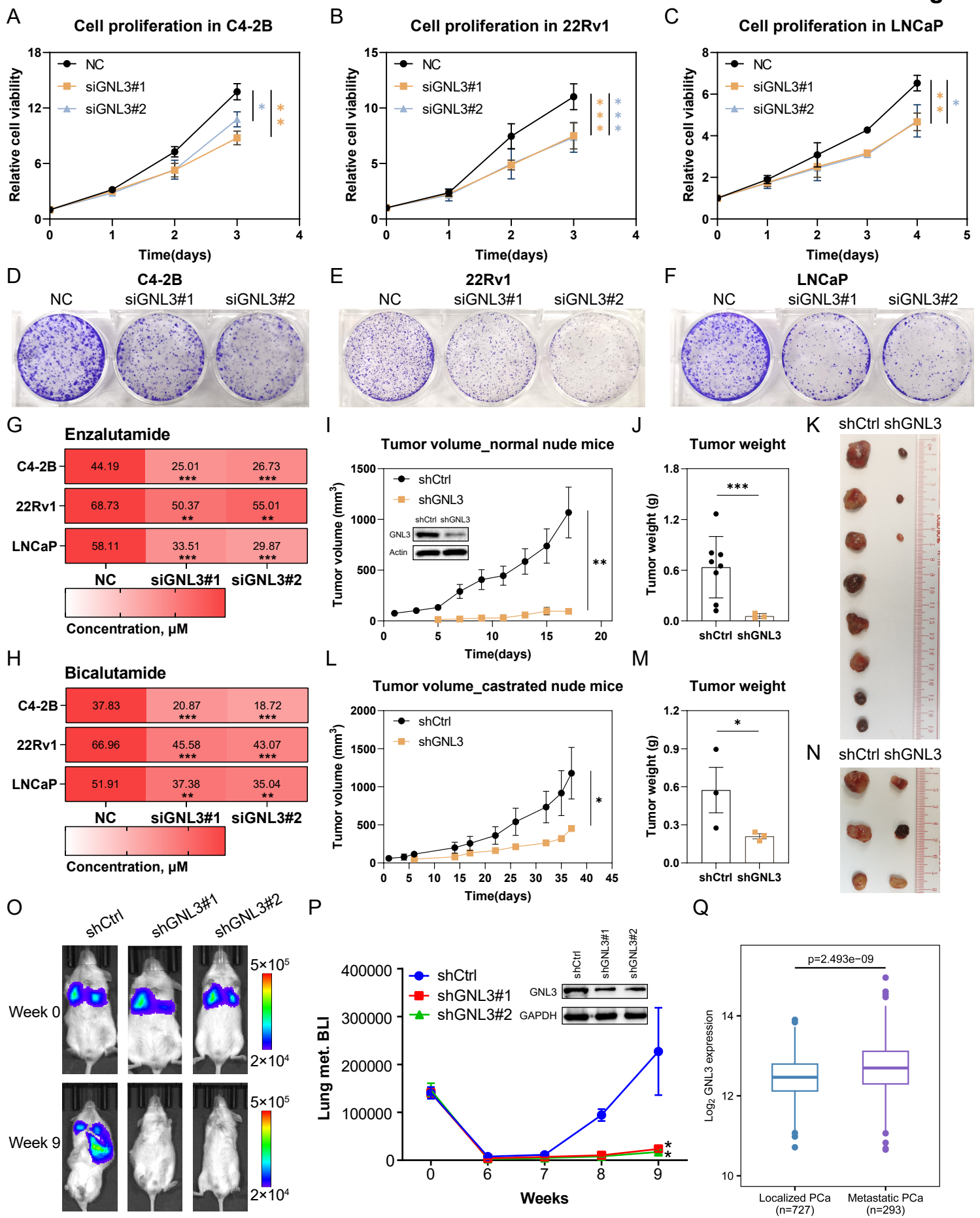

A C4-2B

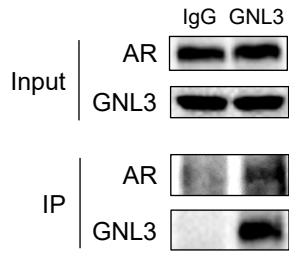

B LNCaP

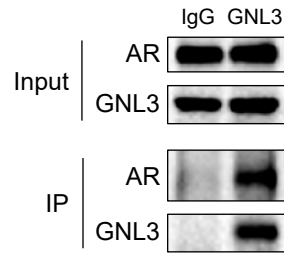

C Proximity Ligation Assay

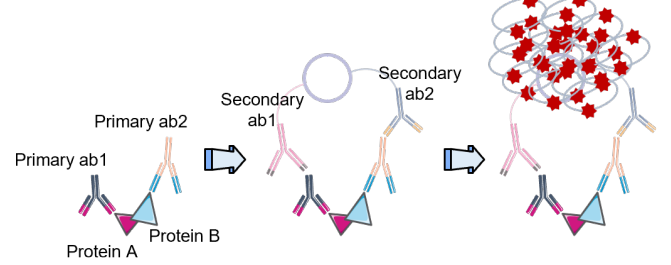

D C4-2B

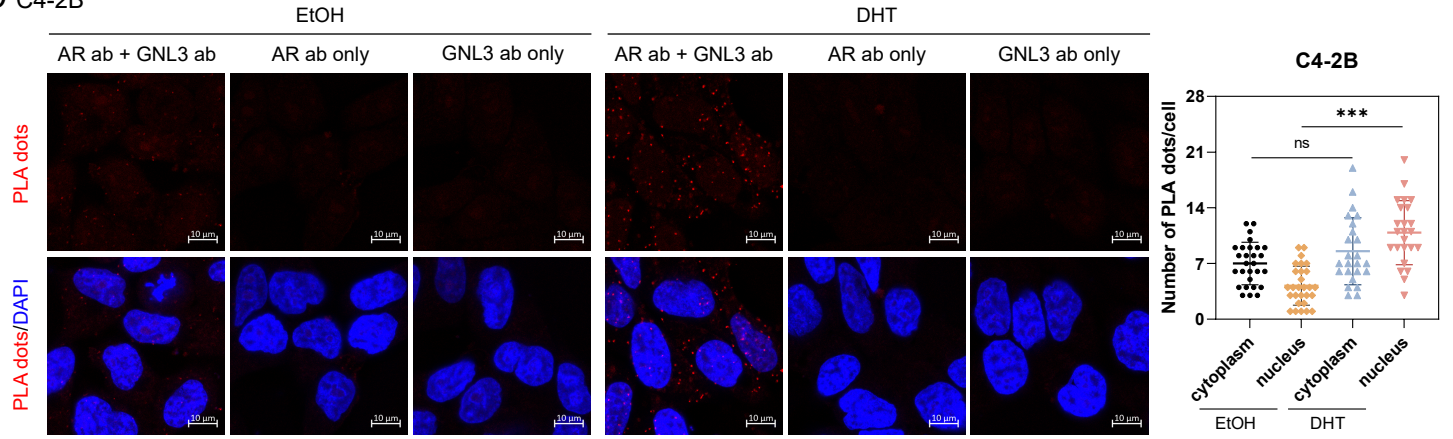

E LNCaP

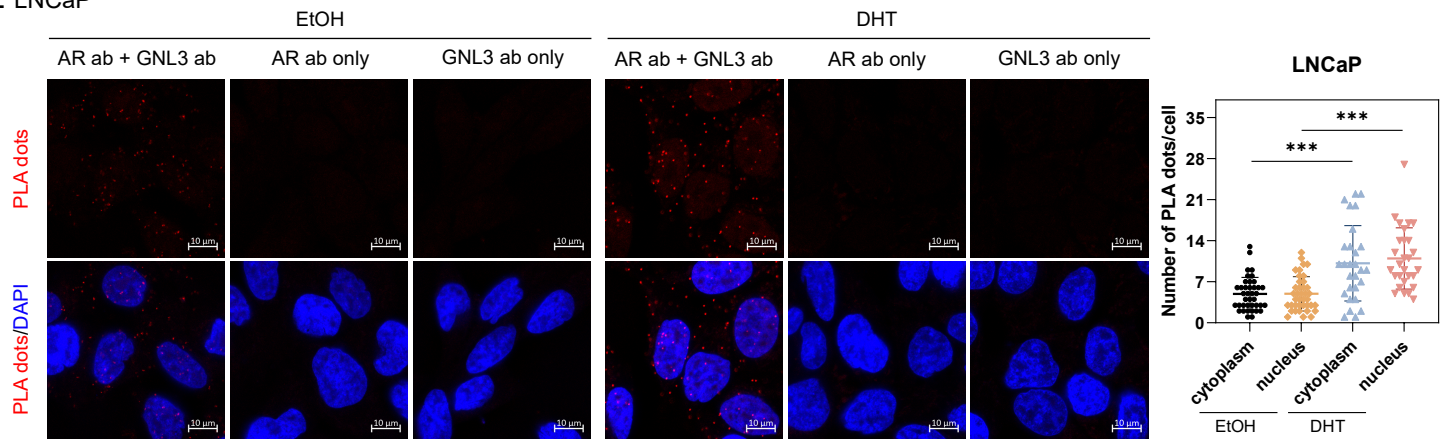

F

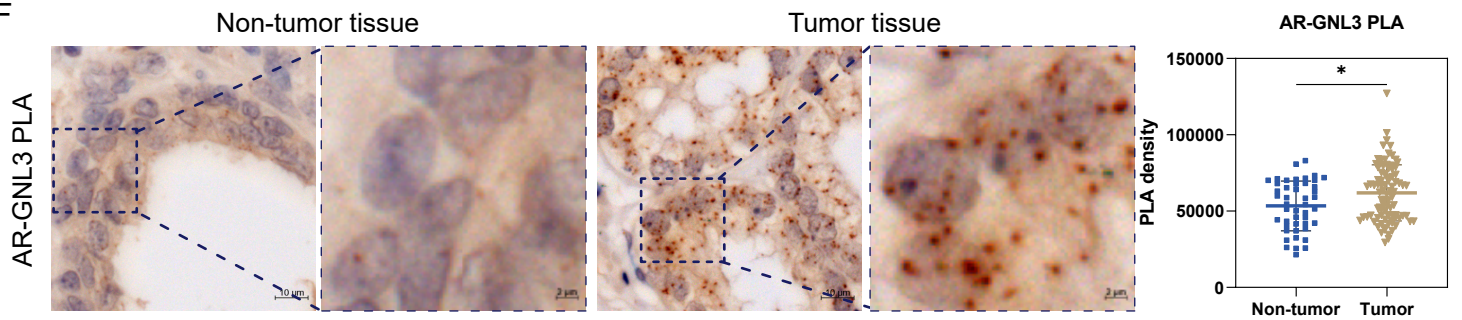

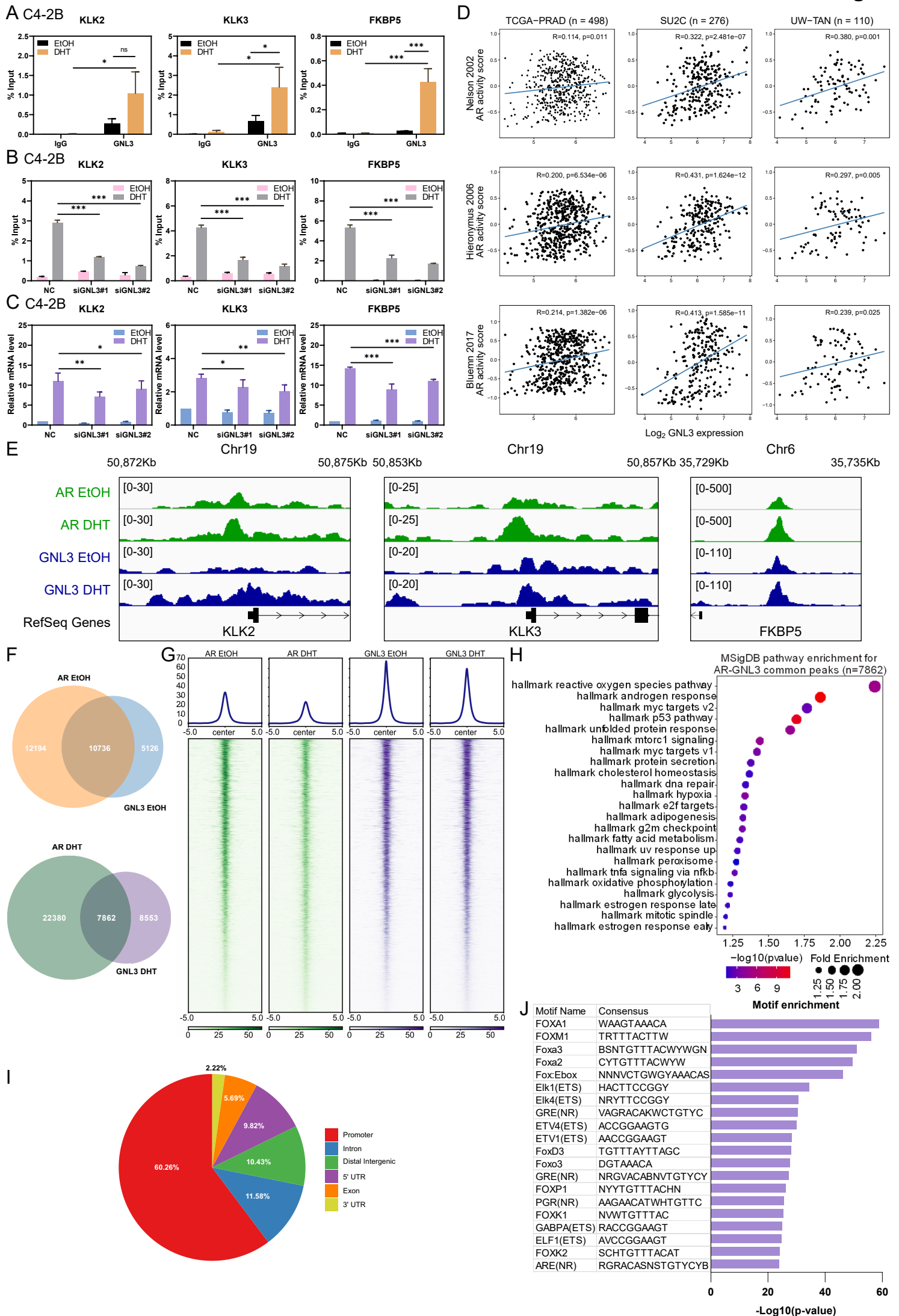

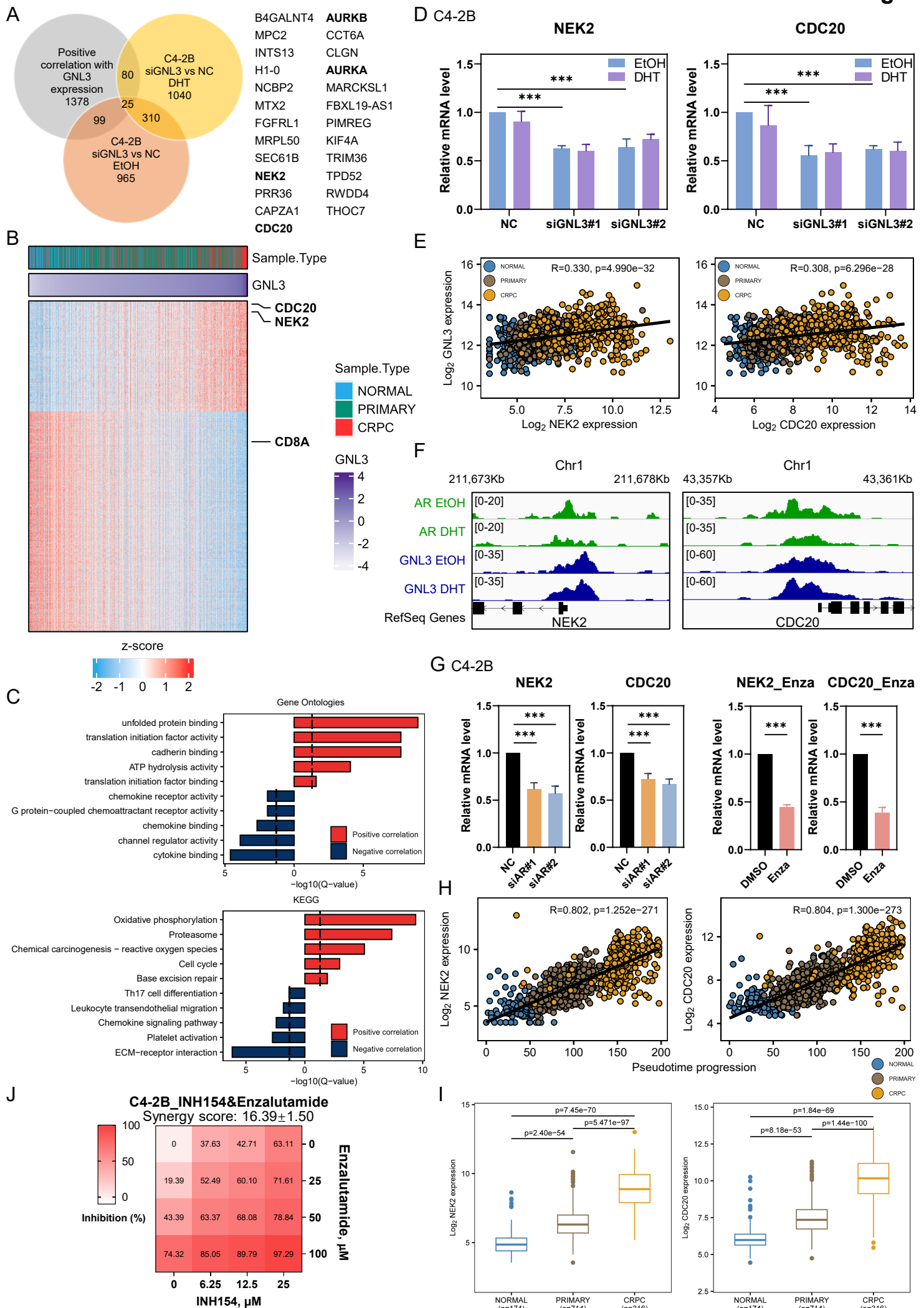

**Figure 6**

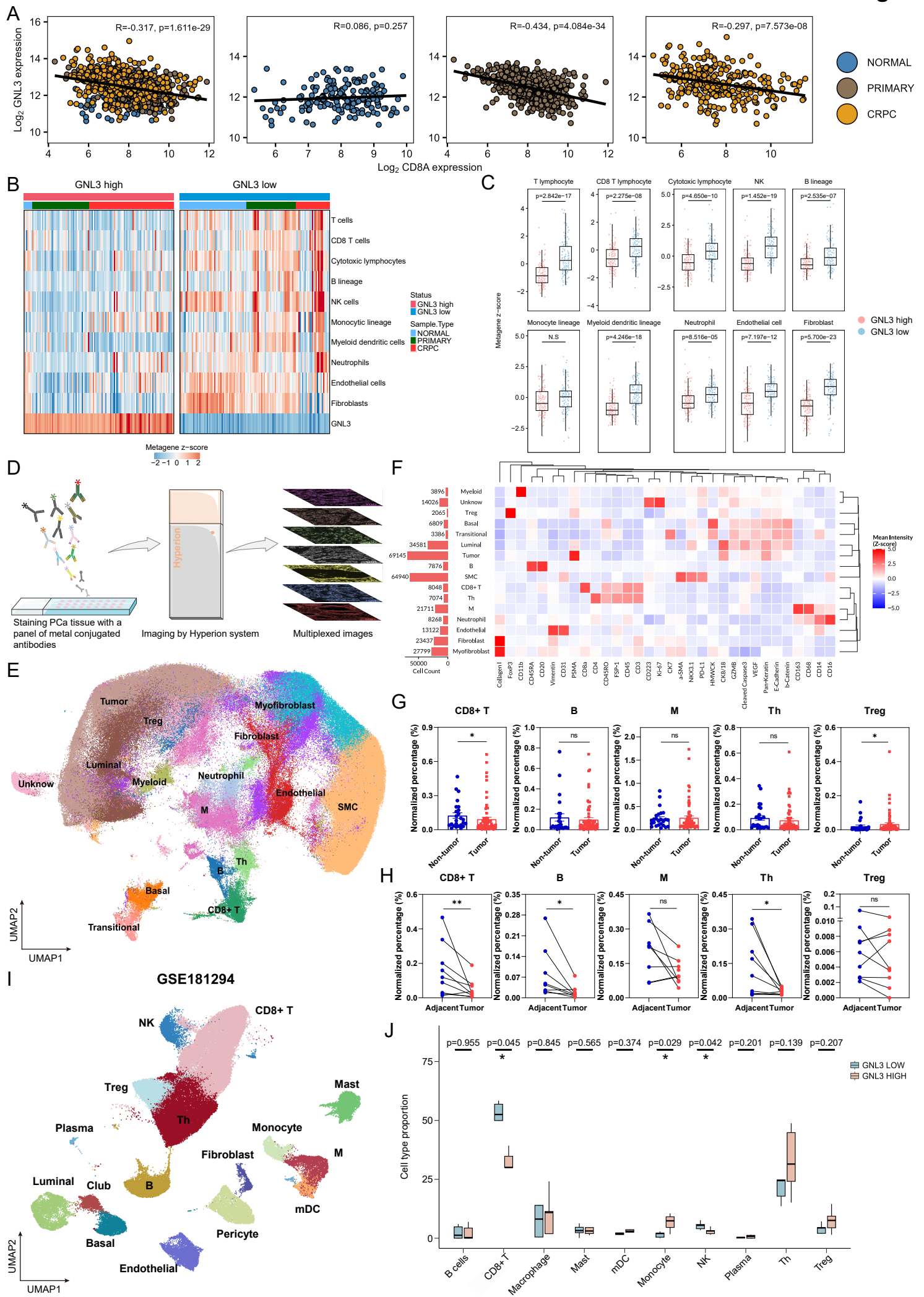

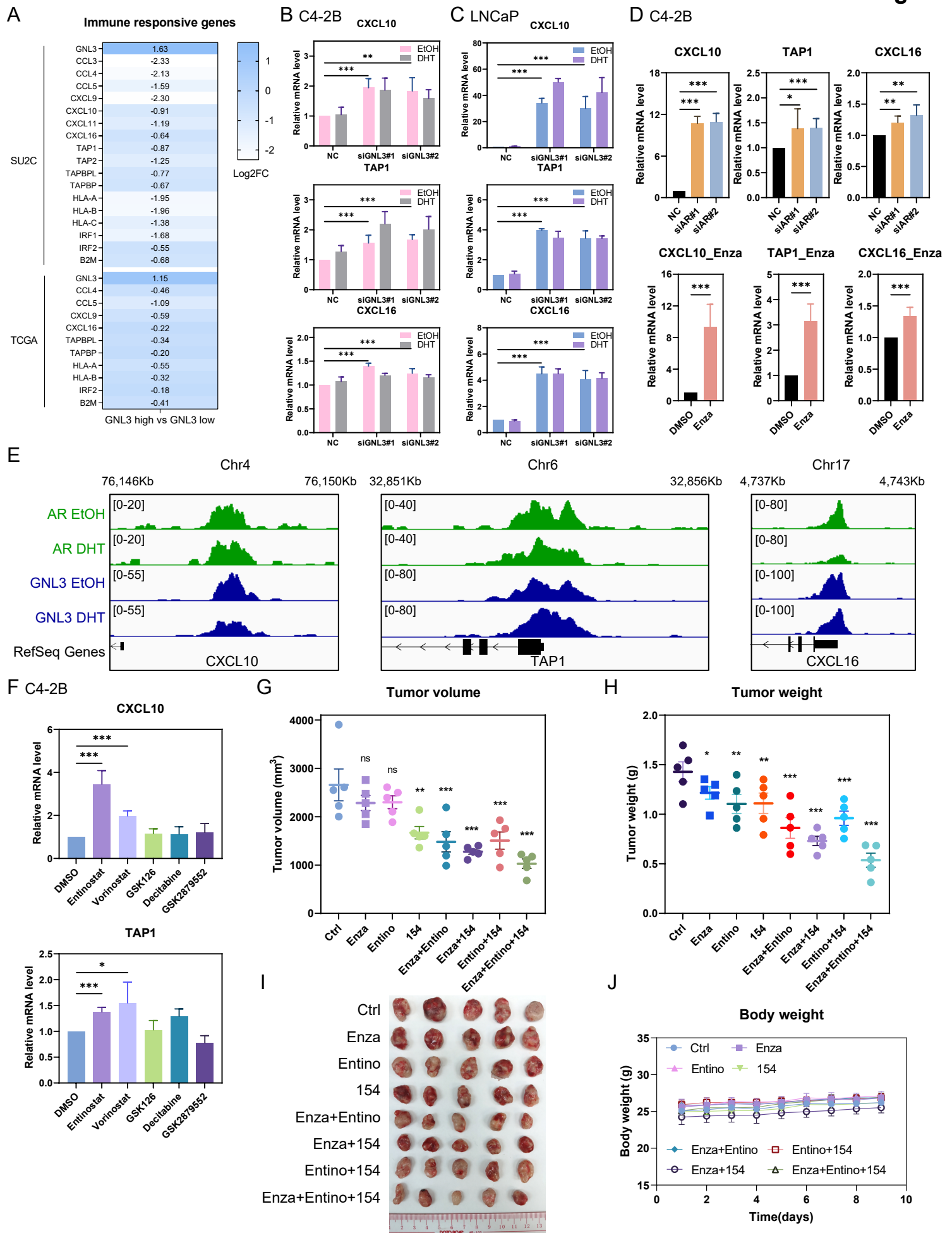

Supplement: Supplementary file 6 — Supporting File 6: advs74573‐sup‐0006‐SuppData.zip. [file ADVS-13-e16411-s001.zip › advs74573-sup-0006-SuppData/Figures.pdf]
